# Advances in MEMS, Optical MEMS, and Nanophotonics Technologies for Volatile Organic Compound Detection and Applications

**DOI:** 10.1002/smsc.202400250

**Published:** 2025-01-15

**Authors:** Dongxiao Li, Hong Zhou, Zhihao Ren, Chengkuo Lee

**Affiliations:** ^1^ Department of Electrical and Computer Engineering National University of Singapore Singapore 117583 Singapore; ^2^ Center for Intelligent Sensors and MEMS National University of Singapore Singapore 117608 Singapore; ^3^ National University of Singapore Suzhou Research Institute (NUSRI) SuZhou 215123 China

**Keywords:** artificial intelligence, electronic nose, health, Internet of Things, photonic nose, sensors, volatile organic compounds

## Abstract

Volatile organic compounds (VOCs) are a class of organic compounds with high vapor pressure and low boiling points, widely present in both natural environments and human activities. VOCs released from various sources not only contribute to environmental pollution but also pose threats to ecosystems and human health. Moreover, some VOCs are considered biomarkers in exhaled breath and can be utilized to identify various diseases. Therefore, monitoring and controlling VOC emissions and concentrations are crucial for safeguarding the environment and human health. In recent years, significant advancements have been achieved in micro‐electromechanical system (MEMS)‐based sensing and optical sensing technologies, offering new avenues for VOC detection. This article provides a comprehensive overview of research progress in MEMS and optical VOC sensors, focusing on their sensing mechanisms and classifications. It then discusses the role of artificial intelligence in enhancing VOC identification and quantification, as well as trends toward sensor miniaturization and intelligence. Furthermore, the article highlights the diverse applications of VOC sensors in medical diagnostics, agricultural food testing, and the Internet of Things. Finally, it emphasizes the opportunities and challenges associated with MEMS and optical VOC sensors, providing valuable insights for practical applications.

## Graphical abstract

Volatile organic compounds (VOCs) are ubiquitous in natural and human environments, posing environmental and health risks. Recent advancements in micro‐electromechanical system and optical sensing offer new possibilities for VOC detection. This article reviews their progress, focusing on mechanisms, artificial intelligence enhancement, and applications in medical diagnostics, agriculture, and Internet of Things. It highlights opportunities and challenges for future applications.

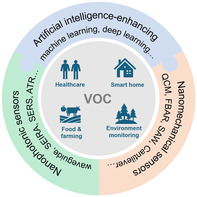

## 1 Introduction

**Figure 1:**
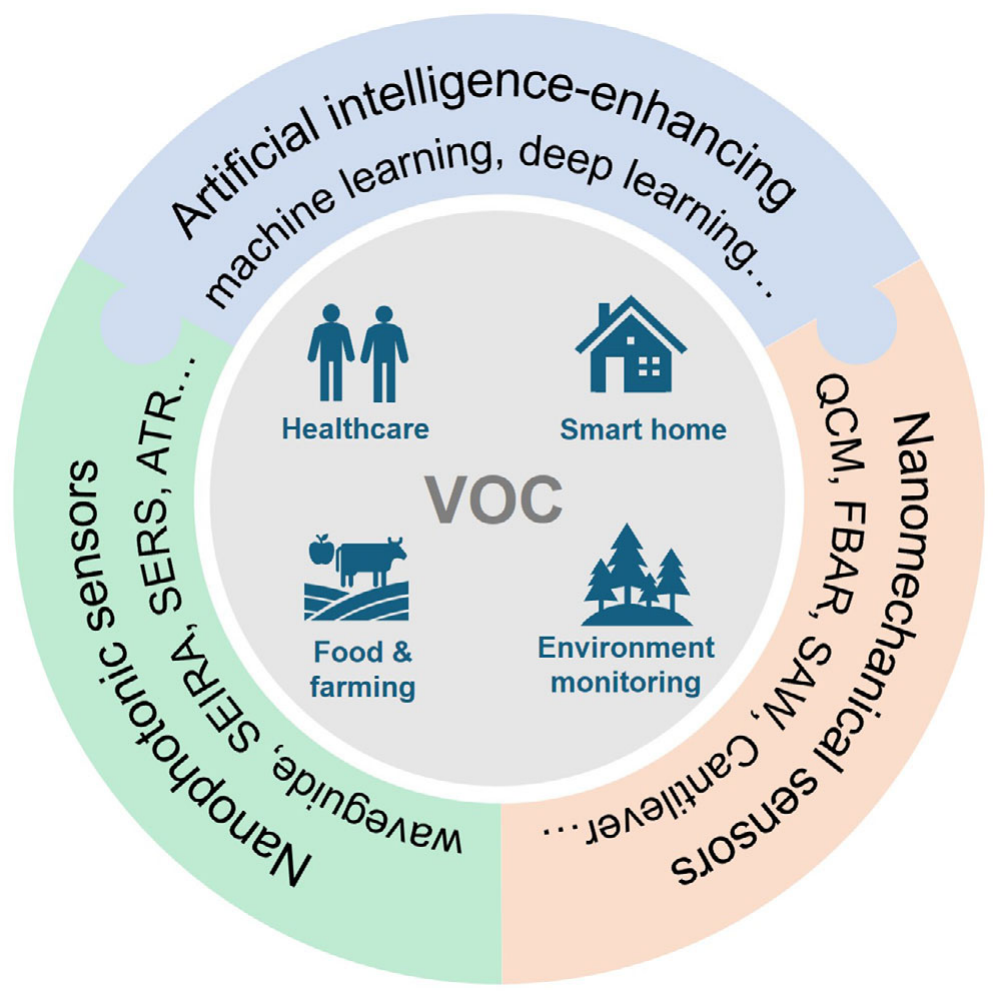
VOC gas sensor applications.

Volatile organic compounds (VOCs) are a class of organic compounds that have high vapor pressure, low boiling point, and are easily volatile under standard conditions.^[^ ^1^ ^]^ The sources of VOCs are typically categorized into natural and anthropogenic. Natural sources include plant emissions, microbial metabolism, combustion, and geological processes.^[^ ^2^ ^]^ Human activities that produce VOCs mainly include industrial production, vehicle exhaust, and the use of household products and building materials.^[^ ^3^ ^]^ Common VOCs include formaldehyde, benzene, xylene, ethanol, and acetone. These compounds primarily exist in the atmosphere as gases but can also dissolve in water or adsorb onto solid surfaces.^[^ ^4^ ^]^ The extensive emission of VOCs can negatively impact ecosystems, leading to issues such as water body eutrophication, reduced soil biodiversity, and inhibited vegetation growth.^[^ ^5^ ^]^ Additionally, some VOCs also participate in photochemical reactions, resulting in the formation of pollutants like ozone and fine particulate matter, thus exacerbating air quality problems.^[^ ^6^ ^]^ In addition to their impact on the environment, VOCs also pose significant risks to human health.^[^ ^7^ ^]^ Most VOCs are volatile and toxic, and they can enter the human body through respiratory tract, skin contact, and food and water intake. Long‐term exposure to high concentrations of VOCs can lead to a range of health problems, including respiratory disease, damage to the nervous system, suppression of the immune system, and some types of cancer.^[^ ^8^ ^]^ Notably, some VOCs are endogenous to the human body, present in exhaled breath, sweat, urine, and other bodily fluids. These endogenous VOCs may reflect the body's internal metabolic state and health status, providing valuable insights for clinical diagnosis and disease monitoring.^[^ ^9^, ^10^, ^11^, ^12^ ^]^ Therefore, monitoring and controlling the emissions and concentrations of VOCs is crucial to protect the environment and human health. The development of VOC sensors offers an effective means for real‐time monitoring and control of these compounds. These sensors can be utilized in various scenarios, including health monitoring and medical diagnosis, indoor air quality monitoring, industrial waste gas treatment, automobile exhaust detection, farm management, and food safety (**Figure** **1**).

To accurately monitor and timely control the emissions and concentrations of VOC, it is crucial to develop efficient and sensitive sensors. Gas chromatography–mass spectrometry (GC–MS) is a commonly used method for detecting VOCs due to its high sensitivity and resolution, which allows for precise identification and quantitative analysis of VOC components.^[^ ^13^ ^]^ However, GC–MS requires relatively bulky, power‐consuming, and expensive equipment and well‐trained personnel, making it unsuitable for real‐time monitoring and field applications.^[^ ^14^ ^]^ Therefore, researchers are focused on developing portable VOC sensors to achieve rapid detection and real‐time monitoring of VOCs. The development of VOC sensor technology has primarily concentrated on four areas: electrochemical sensors,^[^ ^15^ ^]^ metal oxide semiconductor sensors,^[^ ^16^ ^]^ micro‐electromechanical system (MEMS) sensors,^[^ ^17^ ^]^ and optical sensors.^[^ ^18^, ^19^ ^]^ Electrochemical sensors detect changes in the concentration of VOC by measuring the charge transfer between them and electrodes and have the advantages of fast response and low cost. However, electrochemical sensors suffer from problems such as zero drift and aging, which limit their service life. Metal oxide semiconductor sensors utilize the interaction between VOCs and metal oxide surfaces to change their conductive properties. These sensor are suitable for low‐cost mass production but often require high operating temperatures and are high sensitivity to gases and external humidity.

In recent years, advancements in MEMS and optical sensing technologies have significantly improved VOC detection.^[^ ^20^, ^21^, ^22^, ^23^ ^]^ Among them, MEMS sensors achieve detection by monitoring the mass changes between VOCs and micromechanical structures and have extremely high sensitivity and response speed.^[^ ^24^ ^]^ Existing research indicates that MEMS sensors based on mass load can detect mass changes at the nanogram level.^[^ ^25^ ^]^ Due to their high sensitivity, this sensing technology is widely applied in various fields, including biosensing, chemical sensing, and gas sensing.^[^ ^17^, ^26^, ^27^, ^28^ ^]^ Optical sensors utilize the absorption or scattering of light of specific wavelengths by VOCs to achieve detection, which has the advantage of noncontact and real‐time detection.^[^ ^29^ ^]^ However, optical sensors also face some challenges such as complex optical systems, high equipment costs, and susceptibility to environmental light interference. Therefore, further simplifying optical testing systems and enhancing the sensitivity, stability and practicality of the sensor are crucial research directions. Recently, optical sensing technologies based on metasurfaces and waveguides have gradually emerged.^[^ ^30^, ^31^, ^32^, ^33^ ^]^ Metasurfaces, with their unique wavefront manipulation capabilities and high light–matter interaction efficiency, provide opportunities to miniaturize optical measurement systems and reducing costs. On‐chip waveguide technology also presents a promising approach to miniaturization of measurement systems.^[^ ^34^ ^]^ At present, a series of new optical sensing technologies based on metasurfaces and on‐chip waveguides, such as refractive index (RI) sensors,^[^ ^35^, ^36^ ^]^ surface‐enhanced infrared absorption spectroscopy (SEIRA),^[^ ^37^, ^38^, ^39^, ^40^ ^]^ and surface‐enhanced Raman spectroscopy (SERS),^[^ ^41^, ^42^ ^]^ have been developed and are widely used in analyte detection.

This article reviews the field of MEMS and optical‐related VOC sensors, focusing on the main research topics and directions. First, the sensing mechanisms and classifications of MEMS and optical methods are introduced. Then, the latest progress of each technology in VOC sensing applications is introduced one by one. Next, the latest trends in miniaturization and intelligence of VOC sensors are discussed. Finally, some selected application areas are introduced, such as health detection and medical diagnosis, agricultural and food detection, and the application of VOC sensors in the Internet of Things (IoT). At the same time, the opportunities and challenges of MEMS and optical sensors are highlighted, and important insights are provided for further practical applications.

## 2 Sensing Mechanism and Sensor Classification

In this section, we focus on reviewing the VOC sensing mechanisms and their classification based on MEMS and optical methods. Other sensing methods, such as electrochemical sensors, metal oxide semiconductor sensors, photoionization detectors, and thermal sensors, can be found described in more detail in relevant review articles.

### 2.1 Sensing Mechanism

**Figure 2:**
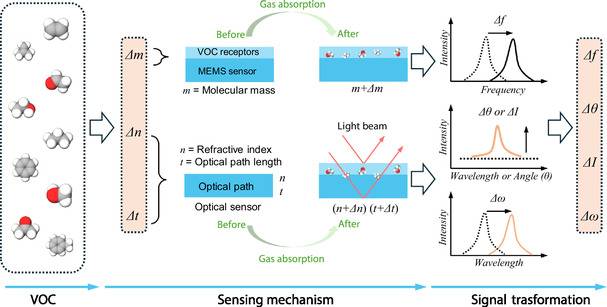
VOC sensing mechanisms and their classification based on MEMS and optical methods.

This review focuses on MEMS resonators and optical methods for VOC sensing. First, MEMS resonators detect VOCs using a gravimetric approach.^[^ ^17^, ^43^ ^]^ However, most MEMS resonators themselves lack the ability to adsorb VOCs. Therefore, in the sensing process, VOC receptors (also known as selectors, sensitizers, functional films, or molecular enrichment membranes) are typically used to capture or adsorb VOC molecules.^[^ ^2^ ^]^ Common VOC receptors include thin films (such as polymers),^[^ ^44^, ^45^, ^46^, ^47^, ^48^, ^49^, ^50^, ^51^, ^52^, ^53^ ^]^ low‐dimensional materials (such as nanotubes and black phosphorus),^[^ ^54^, ^55^, ^56^, ^57^, ^58^, ^59^, ^60^, ^61^, ^62^, ^63^, ^64^ ^]^ porous matrices (such as metal–organic frameworks (MOFs)),^[^ ^18^, ^65^, ^66^, ^67^, ^68^, ^69^, ^70^, ^71^, ^72^, ^73^, ^74^ ^]^ self‐assembled monolayers (SAMs),^[^ ^75^, ^76^, ^77^, ^78^, ^79^, ^80^, ^81^ ^]^ molecularly imprinted polymers (MIPs),^[^ ^82^, ^83^, ^84^, ^85^, ^86^, ^87^, ^88^ ^]^ and metal oxide nanostructures.^[^ ^89^, ^90^, ^91^, ^92^, ^93^, ^94^, ^95^, ^96^ ^]^ These receptors generally offer two adsorption modes: chemisorption and physisorption. Chemisorption is based on intermolecular interactions, primarily determined by the chemical structures of the sensing material and the analyte.^[^ ^97^ ^]^ Physisorption typically involves structural adsorption or van der Waals adsorption, mainly related to the porosity and specific surface area of the sensing material.^[^ ^98^ ^]^ Detailed descriptions of VOC receptors can be found in other reviews.^[^ ^99^, ^100^ ^]^ Through the action of these receptors, VOCs are captured on the surface of the micromechanical structure. The captured VOC molecules alter the mass or elastic coefficient of the micromechanical structure, thereby affecting the resonator's vibration frequency. By measuring changes in the vibration frequency (Δ*f*) of the MEMS resonators, VOC concentration can be detected (**Figure** **2**).

VOC receptors provide a certain specificity for MEMS resonators. By selecting appropriate receptor molecules, specific VOC molecules can be sensed. However, the introduction of VOC receptors also has some negative impacts. For instance, some polymer coatings can reduce the quality factor of MEMS resonators, thereby decreasing measurement sensitivity.^[^ ^101^, ^102^ ^]^ Aging and humidity of receptors like SAMs can also affect the sensor's performance.^[^ ^103^ ^]^ Besides these negative impacts, VOC receptors face the challenge of low selectivity. Typically, a single receptor molecule reacts with multiple VOC gases (known as cross‐reactivity). When detecting complex VOC mixtures, a single receptor has difficulty identifying specific VOCs.^[^ ^104^ ^]^ Therefore, the design and modification of receptor molecules are crucial. One method to enhance selectivity for specific VOCs is to use stronger sensor–analyte interactions, such as covalent bonds and coordination bonds. However, stronger binding between the receptor and VOC can affect the desorption of VOC gases and the sensor's reversibility.^[^ ^14^ ^]^ Although higher operating temperatures can accelerate VOC desorption, they may also lead to irreversible changes in the receptor molecules.^[^ ^105^ ^]^

In recent years, the combination of sensor arrays and advanced data analysis methods has made cross‐reactivity less of an issue.^[^ ^106^ ^]^ For example, loading different receptor molecules onto each sensor in an array mimics the human olfactory system. In this case, each sensor exhibits sufficient diversity to provide different responses to any given VOC in a mixture. The combined responses of different sensors can be used to establish analyte‐specific response patterns (“electronic fingerprints”). By using different response patterns and incorporating classification methods or pattern recognition algorithms, different VOC molecules in a mixture can be identified. This method is commonly referred to as an “electronic nose.”^[^ ^107^, ^108^, ^109^ ^]^ Therefore, in terms of VOC molecule identification, the approach of utilizing cross‐reactivity is more desirable than enhancing selectivity. Currently, sensor arrays based on cross‐reactivity have been successfully used in various applications, including disease detection and food monitoring.^[^ ^110^, ^111^ ^]^

Another method for detecting VOCs discussed in this review is optical sensors. Optical sensors typically relate to changes in volume/thickness/concentration and/or optical properties (such as RI and polarization) caused by the adsorption of VOCs on the sensing layer.^[^ ^112^ ^]^ Here, we illustrate the basic principles of optical sensors using RI as an example (Figure 2). The RI generally comprises two parts: the real part (*n*) and the imaginary part (*k*). The real part of the RI describes the phase velocity of light in the medium (i.e., the speed at which light propagates), which determines the refraction and reflection behavior of light at different medium interfaces. In optical sensors, changes in the real part of the RI can be measured to detect the presence and concentration of substances in the medium. For example, surface plasmon resonance (SPR) sensors detect the adsorption of biomolecules or chemicals by measuring changes in the real part of the RI on a metal surface.^[^ ^113^ ^]^ In SPR sensors, the detection signal associated with changes in the RI is manifested as shifts in the resonance angle (Δ*θ*) or resonance wavelength (Δ*ω*).

The imaginary part of the RI (commonly referred to as the extinction coefficient) describes the absorption of light by the medium, reflecting the energy loss as light propagates through the medium. By measuring changes in light absorption, specific substances in the medium can be detected. For example, in the infrared (IR) spectrum, the imaginary part of the RI corresponds to the characteristic absorption of different molecules at specific wavelengths, known as fingerprint absorption spectra.^[^ ^114^ ^]^ Infrared spectroscopy relies on these fingerprint absorptions to identify various VOC molecules. However, traditional IR spectroscopy techniques are limited by the Beer–Lambert law,^[^ ^115^ ^]^ which is described as follows:

(1) A=εcl

where *A* is the absorbance, *ε* is the molar absorptivity (also known as molar extinction coefficient), *c* is the concentration of the substance, and l is the path length of the light through the substance. To detect low concentrations of VOC gases, a longer optical path is usually required, which results in a larger sensor device. To address this issue, subwavelength metasurface sensing methods have been proposed.^[^ ^40^ ^]^ Subwavelength metasurfaces compress the IR wavelength to the nanoscale, bridging the gap between IR wavelengths (micrometer scale) and molecular sizes (nanometer scale). This strategy not only enhances sensing sensitivity but also significantly reduces the optical path length. Therefore, this review focuses on novel VOC sensing technologies based on metasurfaces or plasmonics.

Quantifying different VOC molecules in optical sensors depends on changes in their volume/thickness/concentration (Figure 2). For instance, the higher the concentration of VOC gas molecules, the stronger the detection signal. However, identifying different VOC molecules solely based on changes in resonance wavelength or resonance angle can be challenging. Therefore, additional VOC receptors are often required for these sensors to enhance their identification capabilities. It is worth noting that Raman spectroscopy and IR spectroscopy inherently include characteristic absorption peaks of VOC molecules. These absorption peaks can be used to identify different VOC molecules without the need for functional films. It should be noted that the introduction of functional films can adsorb more VOC gas molecules, thereby enhancing the sensitivity of Raman and IR spectroscopy. Thus, these analytical techniques are often combined with functional films for quantitative and analytical detection of VOCs.

### 2.2 Sensor Classification

**Figure 3:**
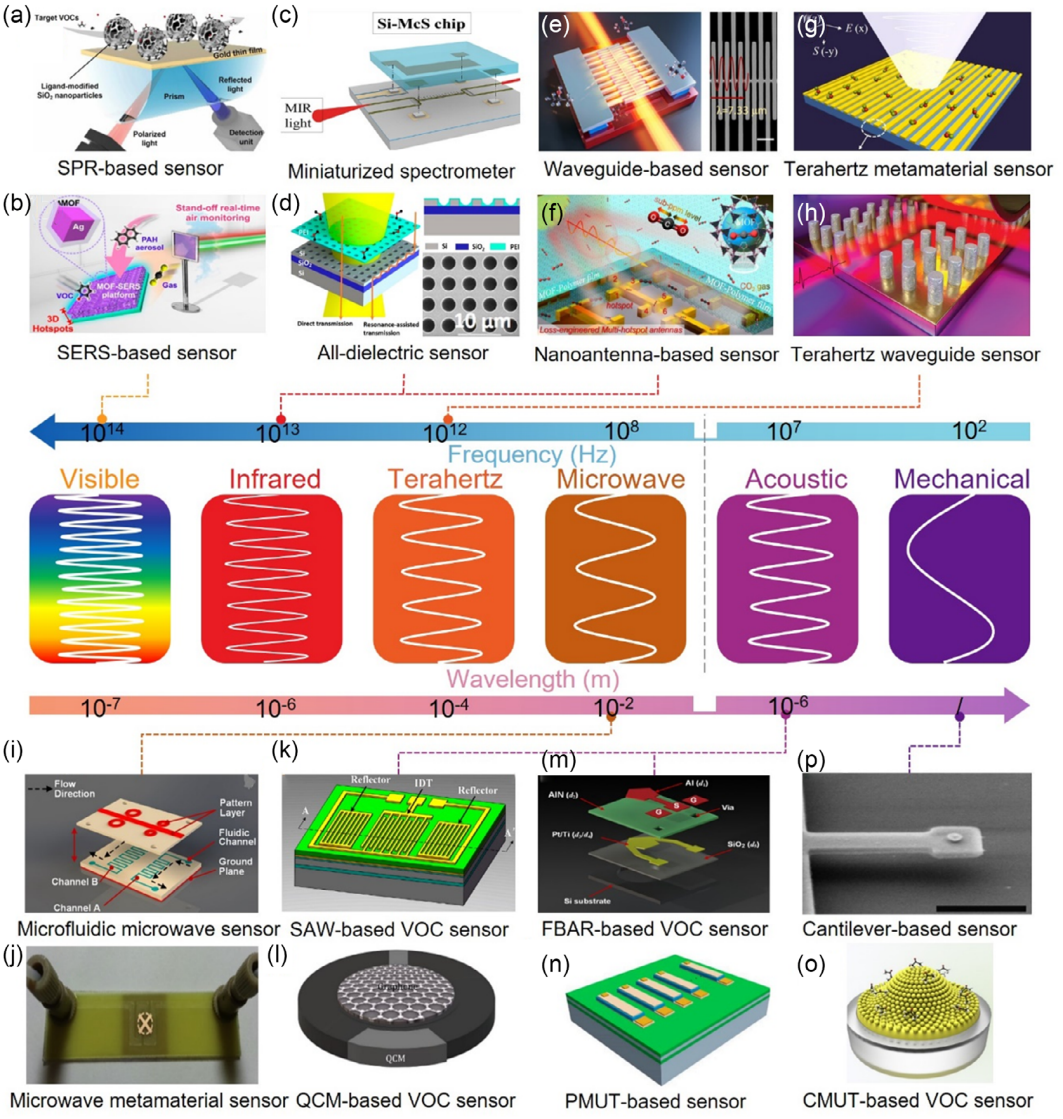
Spectrum of resonance‐based electromagnetic/acoustic/mechanical device for VOC detection. The vertical dashed line in the middle panel distinguishes between devices based on electromagnetic waves (e.g., optical sensors) and those based on mechanical waves (e.g., acoustic wave sensors and mechanical vibration sensors). a) SPR‐based sensors. Reproduced with permission.^[^ ^124^ ^]^ Copyright 2022, Elsevier. b) SERS‐based sensors. Reproduced with permission.^[^ ^125^ ^]^ Copyright 2019, American Chemical Society. c) Infrared miniaturized spectrometer. Reproduced with permission.^[^ ^126^ ^]^ Copyright 2022, American Chemical Society. d) All‐dielectric metasurfaces using guided resonance. Reproduced with permission.^[^ ^127^ ^]^ Copyright 2018, American Chemical Society. e) Waveguide‐based sensors. Reproduced with permission.^[^ ^128^ ^]^ Copyright 2022, American Chemical Society. f) Nanoantenna‐based sensors. Reproduced with permission.^[^ ^129^ ^]^ Copyright 2022, Springer Nature. g) Terahertz metamaterial sensors. Reproduced with permission.^[^ ^131^ ^]^ Copyright 2020, Springer Nature. h) Terahertz waveguide sensors. Reproduced with permission.^[^ ^130^ ^]^ Copyright 2020, John Wiley and Sons. i) Microfluidic microwave sensors. Reproduced with permission.^[^ ^132^ ^]^ Copyright 2018, Springer Nature. j) Microwave metamaterial sensors. Reproduced with permission.^[^ ^133^ ^]^ Copyright 2016, Elsevier. k) SAW‐based VOC sensors. Reproduced with permission.^[^ ^120^ ^]^ Copyright 2018, AIP Publishing. l) QCM‐based VOC sensors. Reproduced with permission.^[^ ^134^ ^]^ Copyright 2021, MDPI. m) FBAR‐based VOC sensors. Reproduced with permission.^[^ ^135^ ^]^ Copyright 2018, American Chemical Society. n) PMUT‐based sensors. Reproduced with permission.^[^ ^136^ ^]^ Copyright 2018, MDPI. o) CMUT‐based VOC sensors. Reproduced with permission.^[^ ^137^ ^]^ Copyright 2018, Elsevier. p) Mechanical cantilever‐based sensors. Reproduced with permission.^[^ ^138^ ^]^ Copyright 2004, AIP Publishing.

Sensors are essential tools for modern‐day technology and play a critical role in various industries, from manufacturing to healthcare to environmental protection.^[^ ^116^, ^117^, ^118^ ^]^ Among them, resonator‐based sensors are a powerful toolkit for VOC detection, with distinct working mechanisms that vary based on their operating frequency region. Electromagnetic, acoustic, and mechanical resonators are the three types.^[^ ^119^, ^120^, ^121^, ^122^, ^123^ ^]^ Electromagnetic resonators in the visible and IR regions can trigger oscillations of free electrons on a metal surface, leading to SPR. SPRs are sensitive to changes in the surrounding molecular RI, and frequency drift or angle changes occur in SPR sensors when exposed to VOC (**Figure** **3**a).^[^ ^124^ ^]^ In the visible region, surface‐enhanced Raman is utilized to detect VOC through VOC‐induced changes in the Raman scattering signal (Figure 3b).^[^ ^125^ ^]^ In the IR frequency region, VOCs show distinct vibrational fingerprint modes which can be enhanced through the interaction between the IR resonator and the molecular vibration, a phenomenon known as SEIRA. The SEIRA effect enables the identification of VOC gases based on their IR fingerprints. The material of the IR resonator can be all‐dielectric (Figure 3c,d)^[^ ^126^, ^127^ ^]^ or metallic (Figure 3e,f)^[^ ^128^, ^129^ ^]^ for SEIRA excitation. Metallic resonators excite plasmons, while all‐dielectric SEIRA requires photonic crystal devices. In terahertz (THz) and microwave regions, plasmons are challenging to excite in metals, but VOC can still affect the electrical performance of resonators. Consequently, terahertz and microwave resonators rely mainly on the dielectric interference of VOC gases for detection (Figure 3g–j).^[^ ^130^, ^131^, ^132^, ^133^ ^]^ Acoustic resonators for VOC detection include surface acoustic wave (SAW) resonators (Figure 3k),^[^ ^120^ ^]^ quartz crystal microbalance (QCM) resonators (Figure 3l),^[^ ^134^ ^]^ thin‐film bulk acoustic wave resonators (FBAR, Figure 3m),^[^ ^135^ ^]^ piezoelectric micromachined ultrasonic transducer (PMUT, Figure 3n),^[^ ^136^ ^]^ and capacitive micromechanical ultrasonic transducers (CMUT, Figure 3o).^[^ ^137^ ^]^ These resonators operate on the gravimetric principle, where the frequency shift of the resonator is measured as the detected signal. In addition to acoustic resonators, mechanical resonators for VOC detection include cantilever beam resonators (Figure 3p).^[^ ^138^ ^]^ It is noteworthy that the sensitive detection of gaseous VOCs requires the use of gas‐selective‐capturing materials, which play a crucial role in the process. These materials facilitate the condensation of low‐concentration gases, thereby intensifying the interaction between the resonator and the gas molecules. **Table** **1** compares the key parameters of MEMS and optical VOC sensors to quickly understand the differences between the different sensors.

**Table 1:** Comparison of key parameters based on MEMS and optical VOC sensors.

Classification	Device type	Working frequency	Sensitivity	Advantages	Shortcomings	Device size	Commercially available
MEMS sensor	QCM	kHz–MHz	Low–moderate	Relatively easy to use, low cost, mature technology	Low operating frequency, low detection resolution, large size, and low integration	≈10 mm	Y
	SAW	kHz–MHz	Moderate–high	High sensitivity, easy integration, low cost, wireless control, mature technology	Part of the energy is lost to a BAW, depending on crystal orientations, susceptible to temperature	1–10 mm	Y
	FBAR	GHz	High	Small size, very high sensitivity, compatible with standard CMOS process, high integration	Large noise/signal ratio, difficult signal control and measurement, complex process, high cost, sensitive to many different parameters	<1 mm	N
	Cantilever	kHz	High	Small size, very high sensitivity, compatible with standard CMOS process	Complex manufacturing and packaging process, susceptible to vibration and temperature	<1 mm	N
	CMUT	MHz	Moderate	High operating frequency, easy integration, high sensitivity	Complex manufacturing process, significantly affected by environment, requires bias voltage	1–10 mm	N
	PMUT	kHz–MHz	Low–moderate	Simple manufacturing, easy integration, low cost	Sensitivity slightly lower than other types, significantly affected by environment	1–10 mm	N
Optical sensor	Infrared plasmonic nanoantenna	IR (0.3–30 THz)	High	High sensitivity, tunable, can enhance molecular vibrations, label‐free	High loss, low Q‐factor, environmental (water vapor) sensitivity	≈100 μm	N
	All‐dielectric metamaterial	Optical to IR (0.3–30 THz)	High	Low loss, high Q‐factor, tunable, less environmental sensitivity	Complex design and fabrication, scalability issues	≈100 μm	N
	SERS	Optical (visible to NIR, 400–1100 nm)	Very high	Ultrahigh sensitivity, single‐molecule detection, rapid response	Requires noble metal nanostructures, reproducibility issues	≈10 nm–10 μm	N
	SPR/LSPR	Optical (visible to NIR, 400–1100 nm)	Moderate–high	High sensitivity, real‐time detection, label‐free	Sensitive to environmental changes, requires noble metal coatings	≈100 μm	N
	Waveguide	Microwave to optical (GHz to THz)	Moderate–high	Compact, integrable, low loss, can be made on‐chip	Design complexity, sensitivity can vary	≈1 mm–10 cm	N
	THz and microwave	THz (0.1–10 THz) and microwave (0.3–300 GHz)	Low–moderate	Deep penetration, nondestructive, suitable for a variety of materials	Sensitivity slightly lower than other optical methods, lower resolution, larger device size	≈1–10 cm	N

## 3 MEMS Sensors for VOC Detections

This section will briefly summarize the six most common MEMS devices used for weight sensing, including SAW resonators, QCM, FBAR, cantilever, CMUT, and PMUT. We will focus on the sensing mechanisms of different MEMS devices and their ability to detect a single gas. The application of MEMS sensor arrays and the detection of multiple gases will be discussed in the electronic nose section. These sensors rely on mass loading of the analyte to induce a change in the resonant frequency. Except for CMUT and cantilevers, the key components of the aforementioned MEMS sensors are piezoelectric materials. Common piezoelectric materials include aluminum nitride (AlN),^[^ ^139^, ^140^ ^]^ zinc oxide (ZnO),^[^ ^141^, ^142^ ^]^ barium titanate (BaTiO_3_),^[^ ^143^, ^144^, ^145^ ^]^ lead titanate (PbTiO_3_),^[^ ^146^, ^147^ ^]^ quartz (SiO_2_),^[^ ^148^, ^149^ ^]^ lithium niobate (LiNbO_3_)^[^ ^150^, ^151^ ^]^ and poly(vinylidene fluoride) (PVDF).^[^ ^152^, ^153^ ^]^ Detailed parameters for each piezoelectric material can be obtained in other reviews.^[^ ^154^ ^]^

### 3.1 QCM‐Based VOC Detection

**Figure 4:**
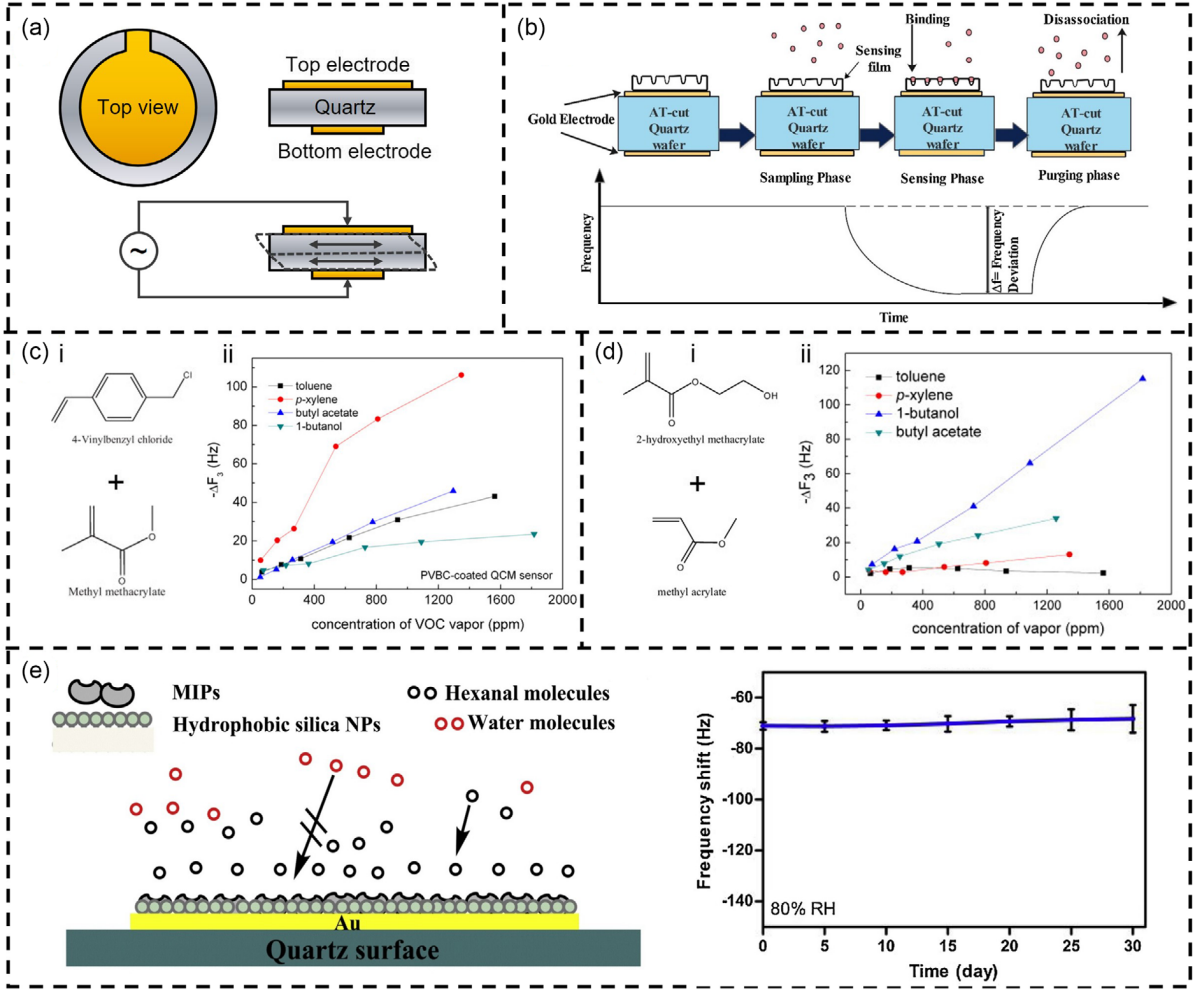
QCM‐based VOC detection. a) The basic structure and working mechanism of QCM. b) Sensing and transduction mechanism of QCM sensor. Reproduced with permission.^[^ ^155^ ^]^ Copyright 2016, Elsevier. c) Selective detection of trace p‐xylene by polymer‐coated QCM sensors. i) The chemical structures of monomers, VBC and MMA. ii) Frequency changes of the QCM sensor under different concentrations of VOCs. Reproduced with permission.^[^ ^52^ ^]^ Copyright 2012, Elsevier. d) Selective detection of trace 1‐butanol by QCM sensor coated with copolymer P(HEMA‐co‐MA). i) The chemical structures of monomers, HEMA and MA. ii) Frequency changes of the QCM sensor under different concentrations of VOCs. Reproduced with permission.^[^ ^169^ ^]^ Copyright 2011, Elsevier. e) Detection of hexanal in humid circumstances using hydrophobic molecularly imprinted polymers composite. Reproduced with permission.^[^ ^172^ ^]^ Copyright 2019, Elsevier.

QCM is a typical bulk acoustic wave (BAW) device, which consists of a quartz crystal and two upper and lower metal electrodes (**Figure** **4**a). The quartz crystal exhibits the piezoelectric effect, causing it to oscillate at its natural resonant frequency when an alternating electric field is applied. This resonant frequency is determined by the mass and geometry of the crystal. The wave mode in QCM is the thickness‐shear mode. This mode of QCM has the advantages of low cost, fast response, and simple structure, and is widely used in the sensing of VOCs in gas and liquid environments. Due to these properties, several QCM modules have been commercialized.

In gas sensing, QCM can be used to detect VOCs in the air. When VOC molecules adsorb onto the QCM surface, the mass of the crystal increases, resulting in a decrease in the resonant frequency. By monitoring the frequency change, the presence and concentration of VOCs can be determined (Figure 4b).^[^ ^155^ ^]^ The relationship between the oscillation frequency in the QCM and the surface mass change can be described by the Sauerbrey equation:^[^ ^156^ ^]^

(2) $$\Delta f=-\frac{2f_{0}^{2}}{A\sqrt{\rho \mu }}\Delta m$$

where *f*_0_ is the fundamental resonance frequency, *A* is the active area of the piezoelectric material, and *ρ* and *μ* are the density and shear modulus of the piezoelectric material, respectively. The sensitivity Δ*f/*Δ*m* of the QCMs can be obtained by deforming the Sauerbrey equation, and it can be observed that the sensitivity is proportional to the square of the resonance frequency. In order to obtain higher sensitivity, the resonant frequency of the QCM needs to be increased. The resonant frequency of the QCM is

(3) $$f_{0}=\frac{v}{2d}$$

where *v* is the acoustic velocity of quartz and *d* is the thickness of the quartz. Therefore, the thinner the quartz crystal, the higher the fundamental frequency of the crystal. However, due to processing precision, the thickness of quartz crystal cannot be as thin as the piezoelectric material in other resonators. Therefore, the maximum frequency of QCM is usually below 10 MHz.

As the quartz crystal itself does not have gas sensing properties, it is difficult for gas molecules to directly combine with smooth noble metal electrodes. Therefore, the surface of the QCM needs to be coated with a gas‐sensitive material film with adsorption properties to enhance the adsorption of the sensor to the target gas.^[^ ^157^, ^158^, ^159^, ^160^, ^161^ ^]^ Currently, various gas‐sensitive materials have been reported, such as polymers, MIP, graphene oxide (GO), and MXene.^[^ ^162^, ^163^, ^164^, ^165^, ^166^ ^]^ Among them, polymer materials are commonly used as QCM coatings due to their diverse chemical groups and relatively easy synthesis.^[^ ^167^ ^]^ Another significant feature of polymer materials is their ease of modification.^[^ ^168^ ^]^ By modifying polymer materials, issues such as low selectivity, low response, and low reproducibility encountered in VOC detection with QCM can be addressed. For example, Fan et al. coated QCM with a combination of poly(4‐vinylbenzyl chloride) (PVBC) and poly(4‐vinylbenzyl chloride‐co‐methyl methacrylate) (PVBC‐co‐MMA), achieving high sensitivity, high selectivity, and high reproducibility in the detection of p‐xylene.^[^ ^52^ ^]^ In their work, the polymers were synthesized via free radical polymerization, and then dissolved in chloroform and spin‐coated onto the QCM to form a thin film. Data showed that the addition of MMA enhanced the sensitivity of the polymer coating to p‐xylene vapor (Figure 4c). With a polymer coating thickness of 119 nm, the QCM sensor achieved a minimum detection limit of 54 ppm for p‐xylene vapor, with a frequency shift of 9.6 Hz. In another report, Fan et al. synthesized a new copolymer, P(HEMA‐co‐MA), using the same method.^[^ ^169^ ^]^ They tested the QCM sensor coated with P(HEMA‐co‐MA) for responses to toluene, p‐xylene, butyl acetate, and 1‐butanol. Compared to other tested VOCs, this copolymer film showed higher sensitivity, stability, and selectivity for 1‐butanol (Figure 4d). With a polymer coating thickness of 116 nm, the QCM sensor achieved a minimum detection limit of 72 ppm for 1‐butanol, with a frequency shift of 4.0 Hz. Additionally, these copolymer design method demonstrated good reproducibility and could be reactivated by releasing the adsorbed vapor in a vacuum.

Gas sensors that operate at room temperature are very attractive because they offer extremely low power consumption. For example, Öztürk et al. synthesized pure and variously Pd‐doped ZnO nanorods on a QCM for room temperature VOC sensors.^[^ ^160^ ^]^ Using electrochemical deposition, the doping concentration ranged from 0 to 2.5 mol%. The results showed that all undoped and Pd‐doped nanorod sensors exhibited the highest sensitivity to xylene. Chen et al. developed a QCM gas sensor based on GO and cuprous oxide nanocomposites using a layer‐by‐layer self‐assembly method to detect trimethylamine (TMA) gas at room temperature.^[^ ^161^ ^]^ The results indicated that the sensor had a detection limit of 230 ppb for TMA. Importantly, the sensor maintained good sensitivity and stability after 60 days. However, in practical applications, QCM sensors need to consider the effects of temperature and humidity on sensing performance. To assess the impact of temperature, a common approach is to integrate a temperature sensor within the sensing chamber to record temperature and frequency changes in real time.^[^ ^159^ ^]^ To eliminate the effects of humidity, developing functional coatings with hydrophobic properties is an effective solution.^[^ ^170^, ^171^ ^]^ For instance, Chen et al. reported a QCM gas sensor modified with hydrophobic MIPs composites for hexanal detection.^[^ ^172^ ^]^ In their work, hydrophobic silica nanoparticles were used to resist the effects of humidity. The results demonstrated that the prepared gas sensor exhibited good sensitivity, selectivity, reversibility, and long‐term stability for hexanal at 80% RH (Figure 4e).

### 3.2 SAW‐Based VOC Detection

**Figure 5:**
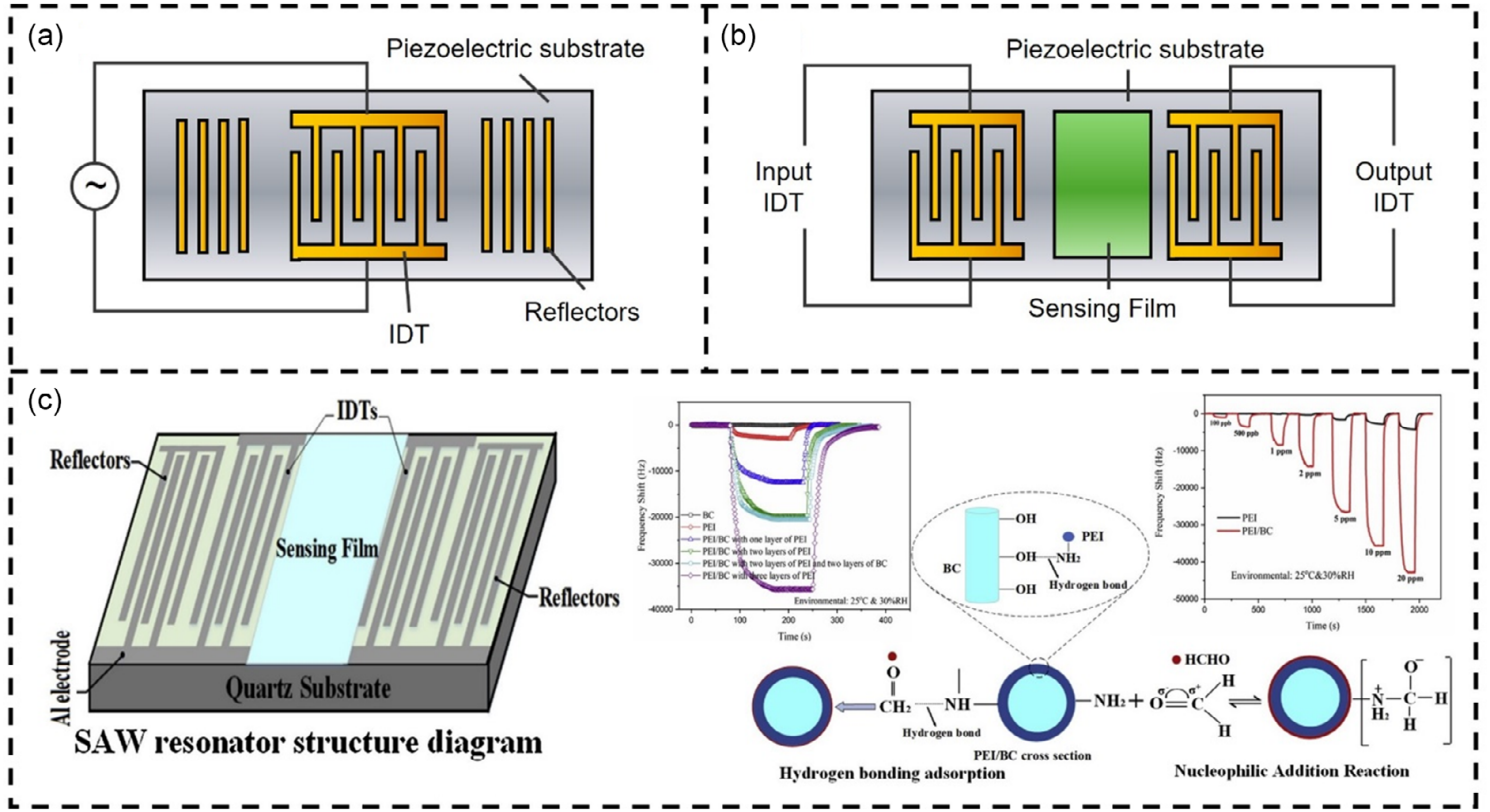
SAW‐based VOC detection. a) The basic structure of a SAW resonator. b) The basic structure of a delay‐line SAW sensor. c) SAW sensor integrating BC and PEI sensing layers for formaldehyde gas detection. Reproduced with permission.^[^ ^195^ ^]^ Copyright 2020, Elsevier.

SAWs are sound waves that propagate along the surface of solid materials. They were first discovered by British scientist Lord Rayleigh in 1885 and are also known as Rayleigh waves.^[^ ^173^ ^]^ SAW devices are composed of piezoelectric materials and periodic comb‐like interdigital transducers (IDT) on their surface. When an alternating voltage is applied to these IDT, the piezoelectric effect causes mechanical deformation in the piezoelectric material, generating acoustic waves. These waves propagate along the surface of the material as SAWs, with the wave direction being perpendicular to the IDT. **Figure** **5**a shows a typical SAW resonator. In this configuration, specially designed grating reflectors with a period of *λ*/2 are used to reflect surface waves back to the IDT for resonance. Figure 5b illustrates a delay‐line type SAW sensor. This type of SAW sensor consists of two sets of IDT: one set serves as the input electrodes and the other set serves as the output electrodes. When a certain voltage is applied to the input electrodes, it generates acoustic waves that propagate from the transmitter to the receiver. A VOC‐sensitive coating or film is applied along the propagation path of the acoustic waves. When VOC molecules in the surrounding environment come into contact with this sensitive coating, adsorption and reaction occur. This adsorption and reaction alter the coating's mass and elastic modulus, thereby affecting the propagation speed and attenuation of the acoustic waves. These changes lead to time delays and amplitude variations in the acoustic signals received by the output transducer compared to the original signals. By measuring the time delays and amplitude variations of these electrical signals, the concentration and type of VOCs can be determined.

The resonant frequency of SAW is

(4) $$f_{0}=\frac{v}{4d}$$

where *d* is the width of finger for IDT and *v* is the sound velocity of the selected piezoelectric material.^[^ ^174^ ^]^ The mass sensitivity of SAW is also described by the Sauerbrey equation. SAW sensors have the advantages of simple structure, high sensitivity, low cost, and miniaturization, and are widely used in gas sensing, biosensing, and chemical sensing.^[^ ^43^, ^175^, ^176^, ^177^, ^178^ ^]^ However, SAW sensors are susceptible to environmental factors such as temperature. The relationship between the resonant frequency (*f*_0_) of SAW and temperature (*T*) can be described by the following formula:

(5) $$\text{TCF}=\frac{1}{f_{0}}\times \frac{df_{0}}{dT}$$

From the above equation, it is clear that the frequency temperature coefficient (TCF) of a SAW device is related to its fundamental frequency. Besides the resonant frequency, the TCF value also depends on the material properties and its cut angle structure. Quartz is the most stable piezoelectric material with a TCF value of 0.^[^ ^179^ ^]^ However, LiTaO_3_ and LiNbO_3_ exhibit frequency shift under temperature fluctuations.^[^ ^180^ ^]^ Therefore, to avoid the impact of temperature fluctuations on frequency shift, it is recommended to choose piezoelectric materials with low TCF. In addition, using an additional SAW sensor as a reference channel can also solve the problem of frequency shift caused by temperature changes during the measurement process.^[^ ^120^ ^]^

Coating a SAW resonator with a sensitizer allows it to interact with target gas molecules, causing a change in acoustic load and shifting the resonance frequency.^[^ ^181^ ^]^ However, a single SAW resonator typically has poor selectivity for multiple target gases. Currently, the mainstream method is to use multiple SAW sensors coated with different sensitizers to detect multiple gas molecules,^[^ ^182^, ^183^, ^184^, ^185^ ^]^ but this also leads to an increase in the number and cost of sensors. To address this issue, Pang et al. successfully demonstrated the simultaneous detection of two analytes using a single SAW resonator coated with a layer of reduced GO (RGO).^[^ ^186^ ^]^ This multianalyte detection capability stems from RGO's different adsorption mechanisms for different gas molecules. Specifically, RGO adsorbs H_2_O through hydrogen bonding, changing the surface density and shear modulus of the RGO film. In contrast, CO_2_ adsorption only changes the surface density of RGO. By decoupling surface density and shear modulus through acoustic load theory, simultaneous detection of CO_2_ and H_2_O is achieved. Results show that the detection errors for CO_2_ and H_2_O with a single SAW resonator are 65 ppm and 1.5% RH, respectively.

The delay‐line type SAW sensor is widely used in sensing applications.^[^ ^187^, ^188^, ^189^, ^190^, ^191^ ^]^ A key issue in SAW sensor design is the choice of the sensitive layer. An appropriate sensitive layer can significantly improve the sensitivity of SAW sensors.^[^ ^192^, ^193^, ^194^ ^]^ For example, Wang et al. used a bilayer nanomembrane of bacterial cellulose (BC) and polyethyleneimine (PEI) as a sensitizer to detect formaldehyde gas (Figure 5c).^[^ ^195^ ^]^ Among them, the BC film has an ultrafine fibrillar and fibrous network structure that provides numerous attachment sites for PEI particles. The addition of BC significantly improved the sensitivity of the SAW formaldehyde gas sensor and reduced the response and recovery times. Furthermore, the sensor employs ST‐cut quartz crystal as the piezoelectric material, with a TCF of 0, making its resonance frequency almost unaffected by temperature. Studies have shown that the SAW sensor exhibits a frequency shift of 35.6 kHz for 10 ppm formaldehyde gas at room temperature and 30% RH, with a detection limit as low as 100 ppb.

### 3.3 FBAR‐Based VOC Detection

**Figure 6:**
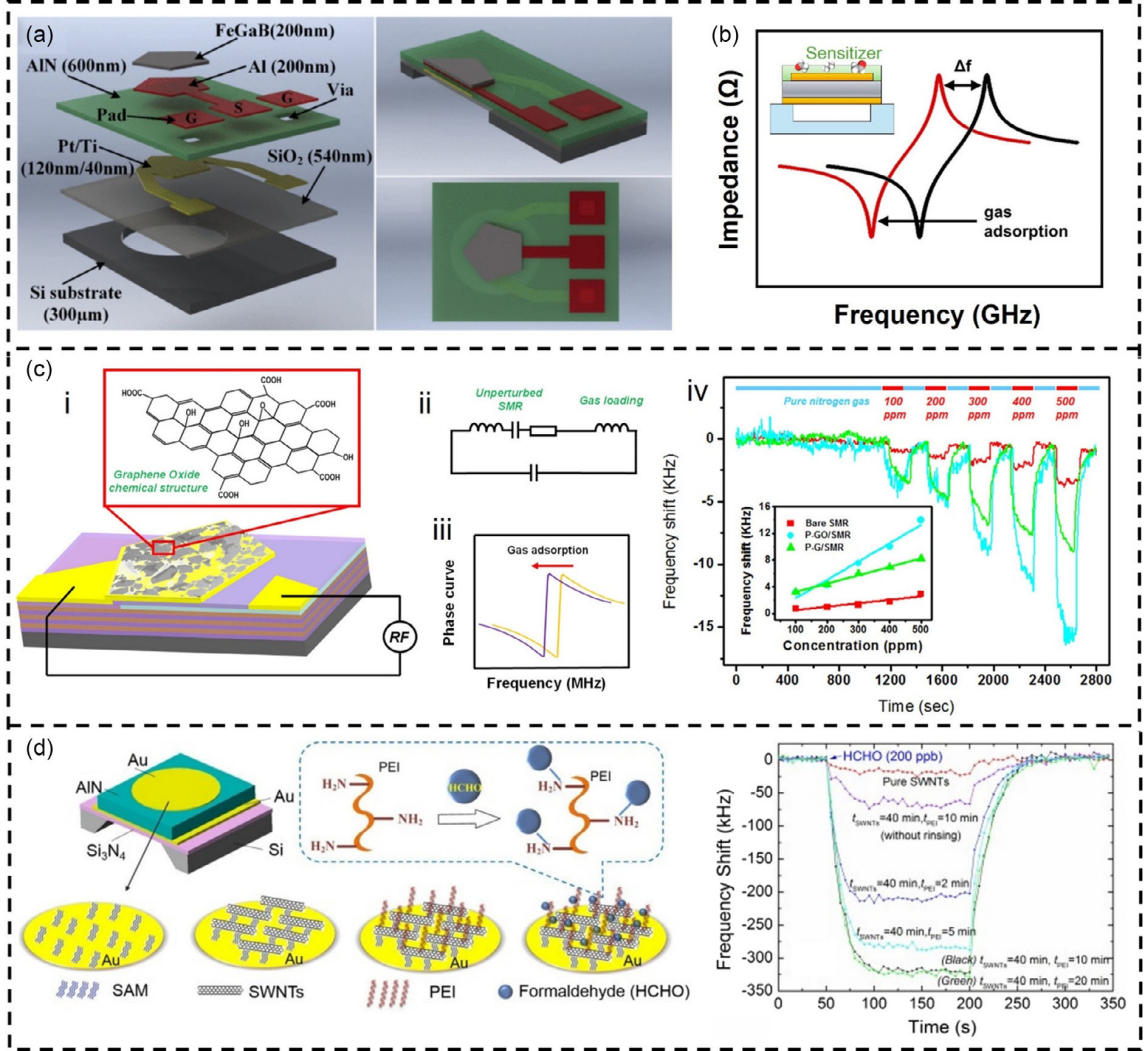
FBAR‐based VOC detection. a) The basic structure of FBAR. Reproduced with permission.^[^ ^135^ ^]^ Copyright 2017, American Chemical Society. b) Detection principle of FBAR gas sensor. Reproduced with permission.^[^ ^204^ ^]^ Copyright 2022, AIP Publishing. c) FBAR coated with GO for NH_3_ sensing. i) FBAR modified with base‐rich GO sheets to enhance surface gas adsorption. ii) The gas sensing butterworth van dyke model. iii) The mass‐loading effect from gas adsorption causes frequency shift of FBAR. iv) FBAR measured the frequency response for different NH_3_ concentrations. Reproduced with permission.^[^ ^205^ ^]^ Copyright 2017, American Chemical Society. d) Formaldehyde sensor based on FBAR. The sensitive membrane is PEI‐modified single‐walled carbon nanotubes. Reproduced with permission.^[^ ^206^ ^]^ Copyright 2018, Elsevier.

In the field of sensing, FBAR has attracted more and more attention due to its advantages such as high sensitivity, low cost, and small size.^[^ ^196^, ^197^, ^198^ ^]^ In many aspects, FBAR is similar to QCM. Therefore, the resonant frequency of FBAR follows the same equation as QCM. **Figure** **6**a depicts a typical FBAR device.^[^ ^135^ ^]^ FBAR consists of two metal electrodes with a very thin piezoelectric material in between. The piezoelectric material of FBAR is usually AlN or ZnO instead of quartz due to their high acoustic velocity and low acoustic loss. As the thickness and size of the piezoelectric film in FBAR are several orders of magnitude smaller than those of QCM, FBAR can achieve very high resonant frequency (from sub‐GHz to tens of GHz) and sensitivity. However, FBAR also has drawbacks, including high manufacturing costs, complex readout circuits/electronics, increased noise, reduced Q‐factor, and lack of good microfluidic functionality.^[^ ^174^ ^]^ These shortcomings lead to the fact that commercial fabrication of FBARs is not as common as QCM and SAW devices.^[^ ^199^ ^]^

FBAR is widely used as a mass sensor in gas sensing applications.^[^ ^200^, ^201^, ^202^, ^203^ ^]^ Figure 6b shows the FBAR gas sensing principle.^[^ ^204^ ^]^ First, a layer of sensitizer is deposited on the surface of the FBAR to selectively adsorb gases. The adsorption of gas molecules increases the mass of the resonator, resulting in a change in the resonant frequency. By detecting the change in resonant frequency, the quantity of gas molecules adsorbed on the surface can be inferred. Zhao et al. coated the surface of an FBAR with a layer of GO sensitive film to detect different concentrations of NH_3_ (Figure 6c).^[^ ^205^ ^]^ In their work, Zhao et al. used oxygen plasma treatment on GO to improve the sensor's sensitivity. The study showed that the sensitivity of the sensor for NH_3_ detection reached ≈27 Hz ppm^−1^. FBARs have also been used for detecting chemical warfare agents. For instance, Yan et al. developed a portable gas sensing instrument for detecting chemical warfare agents.^[^ ^204^ ^]^ This instrument consists of a MEMS‐fabricated micro‐preconcentrator (μPC) and an FBAR gas sensor. The μPC is coated with nanoporous MOF materials to enrich the target, while the FBAR can rapidly detect without the need for additional carrier gas. Experimental results demonstrated that the device has good adsorption and desorption performance for methyl phosphonic acid ester (dimethyl methylphosphonate [DMMP], a simulant for the chemical warfare agent sarin) at a concentration of 1 ppm.

In the field of VOC detection, Song et al. developed a formaldehyde sensor based on a FBAR (Figure 6d).^[^ ^206^ ^]^ In their work, PEI‐modified single‐arm carbon nanotubes were used as the sensitive coating. Due to the mass‐loading effect, the sensitive layer's absorption of formaldehyde resulted in a significant negative frequency shift. The study showed that this sensor could detect formaldehyde at ppb concentrations (limit of detection (LOD) is 24 ppb), with a response time of less than 1 min. Wang et al. utilized the same materials as a sensitive coating to detect formaldehyde gas.^[^ ^102^ ^]^ Unlike previous approaches, carbon nanotubes and PEI were assembled layer by layer on top of the FBAR. This layer‐by‐layer assembly allows for precise tuning of the FBAR's resonant frequency by controlling the number of deposited layers. Additionally, the multilayer films exhibit a random and porous structure, providing a large specific surface area for gas adsorption and diffusion. The results demonstrated that the sensor could easily detect formaldehyde at ppb concentration levels, with a response time of less than 1 min. The gas sensitivity of the FBAR sensor was 1.29–1.90 kHz ppb^−1^, with a detection limit of 24–38 ppb.

### 3.4 Cantilever‐Based VOC Detection

**Figure 7:**
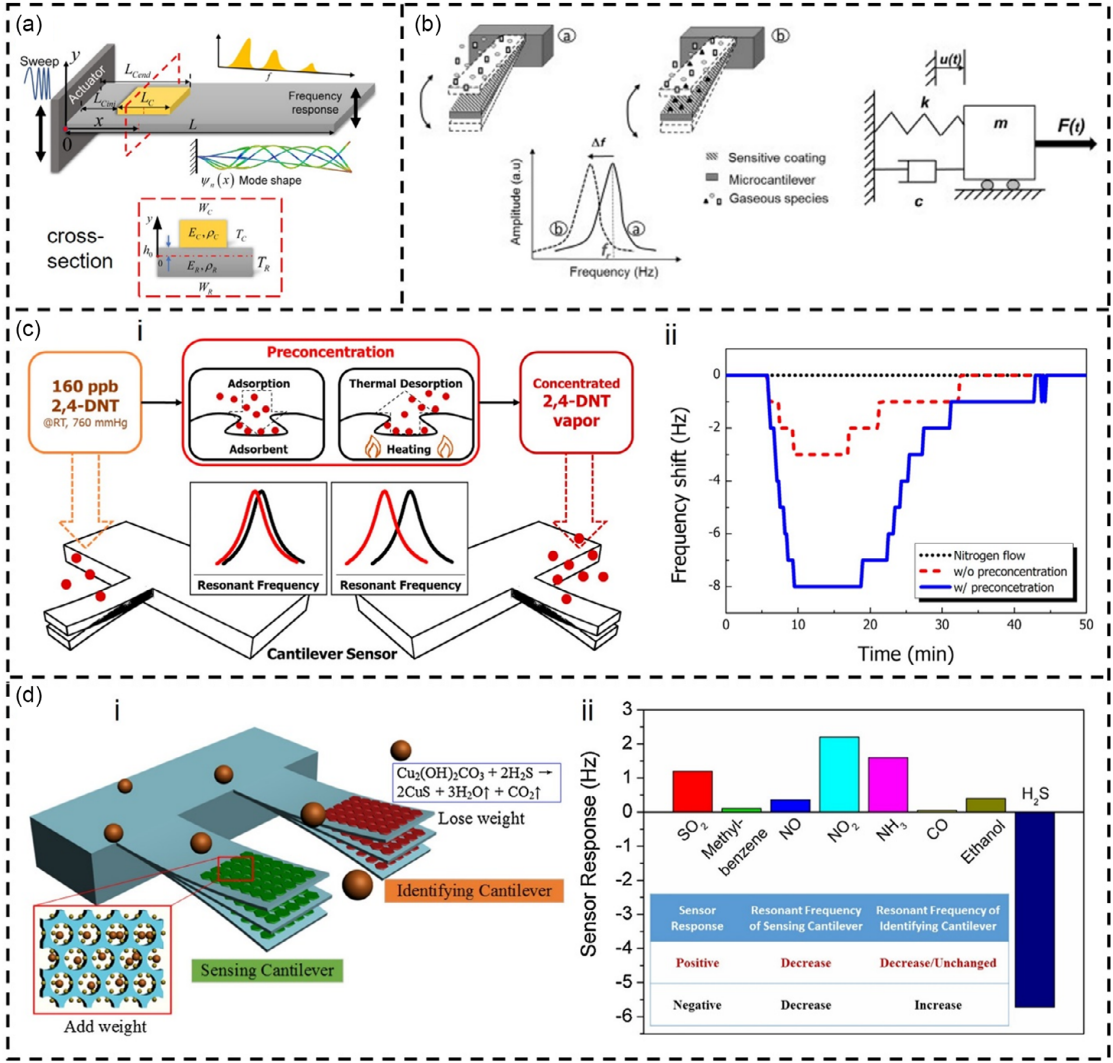
Cantilever ‐based VOC detection. a) The schematic diagram of the cantilever resonator with an adsorbate. Reproduced with permission.^[^ ^207^ ^]^ Copyright 2022, MDPI. b) Resonant microcantilever used as chemical microsensor. Reproduced with permission.^[^ ^216^ ^]^ Copyright 2020, Elsevier. c) i) Functional model of the cantilever‐based artificial olfactory system with sample preconcentration for 2,4‐DNT vapor detection. ii) Relative signal response with different sample preprocessing environments. Reproduced with permission.^[^ ^224^ ^]^ Copyright 2015, MDPI. d) i) Schematic view to illustrate the combined detection to H_2_S with the integrated resonant dual‐microcantilevers combined sensor. ii) Responses of the resonant dual‐microcantilevers combined sensor to various interfering gases. Reproduced with permission.^[^ ^226^ ^]^ Copyright 2020, Elsevier.

The cantilever beam resonator is a MEMS device based on a cantilever beam structure (**Figure** **7**a).^[^ ^207^ ^]^ It typically consists of a cantilever beam that is fixed at one end and free at the other end. By applying an external force to its free end, it induces vibrations. Cantilever sensors originated in the mid‐1990s, initially used as probes for atomic force microscopes.^[^ ^208^ ^]^ In recent years, cantilever‐based sensors have been widely used in the field of biochemical sensing.^[^ ^209^, ^210^, ^211^, ^212^, ^213^, ^214^, ^215^ ^]^ Micromechanical cantilevers have significant advantages such as small size, strong IC compatibility, and can provide chemical sensors with high sensitivity, high integration, miniaturization, mass production, and large‐scale array structures. Cantilever‐based chemical sensors can be divided into two measurement modes: static and dynamic.^[^ ^216^ ^]^ In the static mode, the change in surface stress or bending deflection of the microcantilever sensor is measured when it adsorbs target substances. In the dynamic mode, the absorbed substance can be detected by measuring the decrease in resonant frequency due to mass increase.^[^ ^43^ ^]^ This review focuses on the second mode of operation. There are two commonly used methods for measuring resonant frequency: optical measurement and piezoelectric measurement. Although optical measurement methods offer high accuracy and sensitivity, the complex measurement systems are not suitable for on‐chip integration. In contrast to optical detection systems, piezoelectric microcantilevers provide electrical signals in their output, which can be easily quantified, thereby allowing device miniaturization. Consequently, piezoelectric microcantilever sensor technology has developed rapidly in recent years.^[^ ^217^, ^218^, ^219^ ^]^ The mechanical resonant frequency of the cantilever beam is defined by its dimensions, and the mathematical expression is as follows:

(6) $$f_{0}=0.162\frac{d}{l^{2}}\sqrt{\frac{E}{\rho }}$$

where *d* is the thickness of the cantilever, *l* is the length of the cantilever, *E* is the Young's modulus of the cantilever material, and *ρ* is the density of the cantilever material.^[^ ^220^ ^]^ The relationship between the oscillation frequency of the cantilever beam and the surface mass change (Δ*m*) is as follows:

(7) $$\Delta f=\frac{1}{2}\frac{f_{0}}{m_{\text{eff}}}\Delta m$$

where *Δf* is the frequency shift, *f*_0_ is the initial resonant frequency, and *m*_eff_ is the effective mass of the cantilever. Through the above equations, the mass sensitivity of the integrated microcantilever sensor can be obtained (Δ*f/*Δ*m*). However, the complex manufacturing and packaging processes of cantilever resonators limit their widespread application in VOC sensing.

To sense gas molecules, microcantilevers must be equipped with a gas‐sensitive layer. Polymers can be used as sensitive materials for detecting VOCs because the absorption and desorption of VOCs in polymers are generally completely reversible.^[^ ^221^ ^]^ For example, Shin et al. fabricated a piezoelectric microcantilever transducer based on Pb(Zr, Ti)O_3_ for detecting alcohol (ethanol or methanol) vapors.^[^ ^222^ ^]^ The fundamental resonant frequency of the microcantilever was 26.2 kHz. To enhance gas sensitivity, the surface of the microcantilever was coated with polymethyl methacrylate (PMMA). Research indicates that PMMA‐coated microcantilevers exhibit a linear frequency shift in response to methanol vapor concentrations, with a sensitivity of 0.03 Hz ppm^−1^. However, further studies are needed to optimize the sensing performance. Preconcentrating samples is an effective way to improve sensitivity and detection limits.^[^ ^223^ ^]^ Chae et al. developed a vapor detection system incorporating a micro‐preconcentrator (μPC) and a microcantilever to detect ultralow concentrations of 2,4‐dinitrotoluene (2,4‐DNT) vapor (Figure 7c(i)).^[^ ^224^ ^]^ A specific sequence of 12‐mer peptides was immobilized on the cantilever surface as receptors for 2,4‐DNT vapor, providing selectivity for the cantilever sensor. The μPC was also fabricated using MEMS technology and coated with an adsorbent polymer. The μPC was placed in front of the sensing reaction chamber, where it could accumulate and release the target vapor. Studies demonstrated that this method tripled the signal amplitude (Figure 7c(ii)) and enabled the sensing system to detect 2,4‐DNT concentrations as low as a few 100 ppb.

Unlike conventional organic (polymer) coatings, Kilinc et al. proposed an inorganic molecular coating for cantilever sensors for VOC sensing applications.^[^ ^225^ ^]^ In their work, 1D ZnO nanorods were used as the inorganic molecular coating, and deposited on the cantilever using an electrodeposition method. After electrodepositing the ZnO nanorods, they were hydrothermally etched to form nanotubes. Compared to nanorods, ZnO nanotubes have a larger surface area and more oxygen vacancies, providing higher sensitivity. Studies have shown that the sensitivity of nanotube‐coated microcantilevers was more than 10 times higher than that of nanorod‐coated ones. The sensor's phase stability and detection limit for diethylamine were 0.02° and below 10 ppm, respectively. However, the selectivity of the porous structure was limited. An effective method to improve the cantilever selectivity is to coat multiple cantilever surfaces with multiple adsorption films. Multiple adsorptive films provide various adsorption modes for the target gas, including physical and chemical adsorption. By combining data postprocessing, precise identification of target gases can be achieved. For example, Tang et al. proposed a novel gas sensor integrating dual resonant microcantilevers for identifying and detecting trace amounts of H_2_S.^[^ ^226^ ^]^ The sensor consisted of two integrated resonant microcantilever sensors with different functions (Figure 7d(i)). One sensor was coated with a zeolitic imidazolate framework (ZIF‐8). However, the selectivity of porous ZIF‐8 was limited, so this microcantilever was used only for concentration measurement. The other cantilever sensor was coated with basic copper carbonate (BCC) for H_2_S molecule recognition. BCC can chemically react with H_2_S. However, the reaction between BCC and H_2_S is irreversible, making a separate recognition cantilever unsuitable for H_2_S detection. By combining the two cantilevers, high sensitivity and selective detection of H_2_S were achieved (Figure 7d(ii)). Experimental results showed that the detection limit of the combined sensor can reach 1ppb.

### 3.5 CMUT‐Based VOC Detection

**Figure 8:**
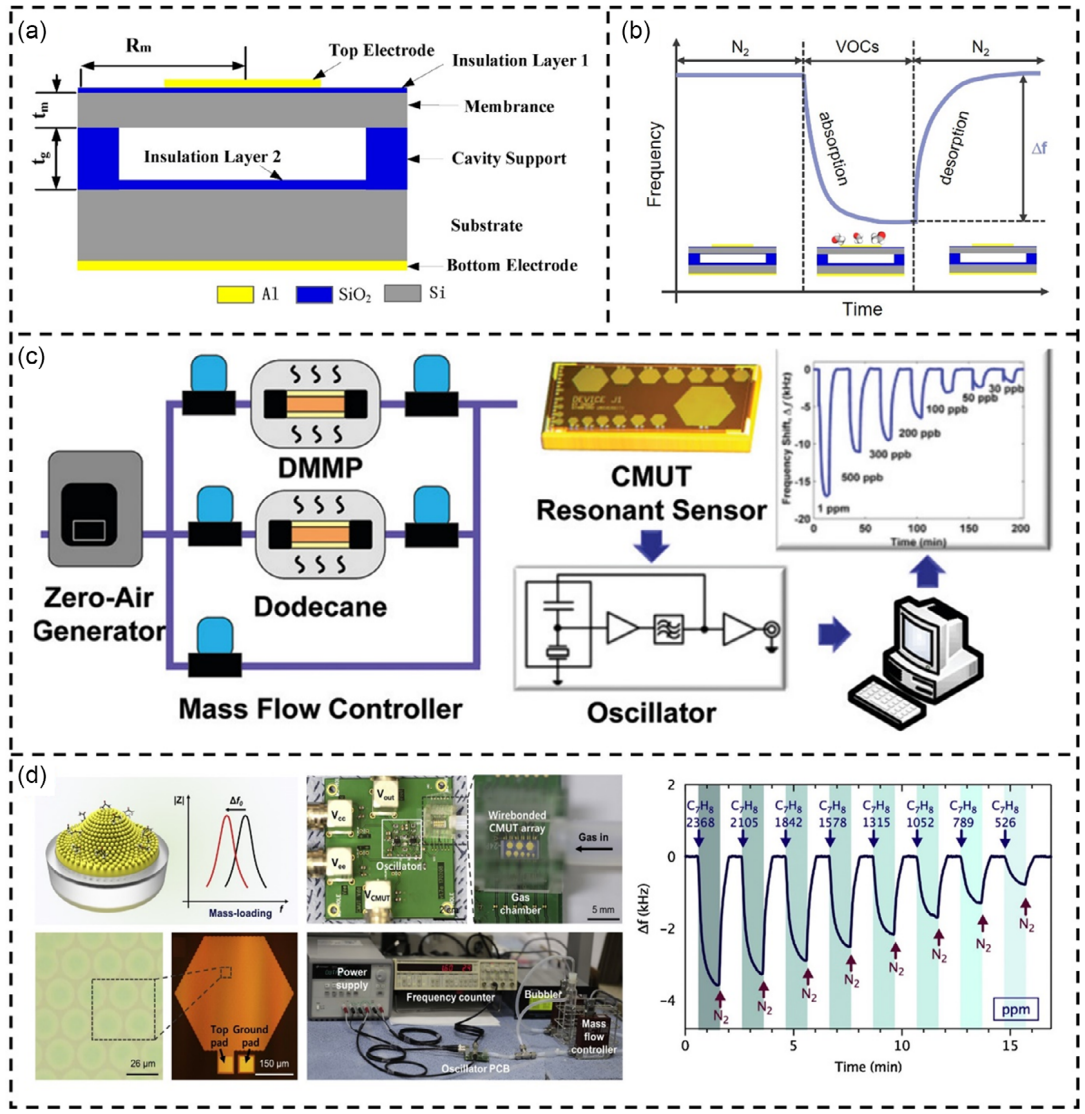
CMUT‐based VOC detection. a) The schematic diagram of CMUT. Reproduced with permission.^[^ ^461^ ^]^ Copyright 2016, MDPI. b) Schematic diagram of CMUT used as VOC sensing principle. c) Chemical vapor detection using a capacitive micromachined ultrasonic transducer. Reproduced with permission.^[^ ^240^ ^]^ Copyright 2011, American Chemical Society. d) CMUT‐based resonant gas sensor array for VOC detection with low operating voltage. Reproduced with permission.^[^ ^137^ ^]^ Copyright 2018, Elsevier.

In recent years, CMUT have emerged as strong contenders for microelectromechanical resonant chemical sensor systems.^[^ ^227^ ^]^ Compared to cantilever beams, CMUT possess inherent performance and manufacturing advantages, making them highly attractive devices for mass sensing applications.^[^ ^228^ ^]^ CMUT were introduced by MI Haller and B.T. Khuri‐Yakub in the mid‐1990s.^[^ ^229^, ^230^ ^]^ Like parallel plate capacitors, CMUT consist of a movable membrane (top electrode), a cavity, and a fixed substrate (bottom electrode) (**Figure** **8**a). When a direct current bias voltage (*V*_DC_) is applied to the electrodes, the membrane is pulled down by electrostatic forces. Subsequently, a small alternating current voltage (*V*_AC_) at a certain frequency is applied to generate alternating electrostatic forces, causing the membrane to vibrate. This enables CMUT to be used as resonant sensors or ultrasonic transducers. Typically, the resonance frequency of a circular CMUT is given by the following equation^[^ ^231^ ^]^:

(8) $$f_{0}=0.47\frac{t}{R^{2}}\sqrt{\frac{E}{\rho \left(1-\nu^{2}\right)}}$$

where *R* and *t* are the radius and the thickness of the plate, respectively. *ρ*, *E*, and *ν* are the density, Young's modulus, and Poisson's ratio of the plate, respectively. Based on this equation, the resonance frequency *f*_0_ is inversely proportional to the density *ρ* of the plate. When CMUT are used as gas sensors, the top membrane is functionalized with sensing materials. Various polymers can be used as sensing materials in gas sensing applications, including polyisobutylene (PIB) and methylated poly(ethylene imine).^[^ ^231^, ^232^ ^]^ When the CMUT gas sensor is exposed to the target gas, the sensing material absorbs the target gas molecules. The adsorption of gas molecules causes a change in the density of the sensor's top plate, which, in turn, causes the sensor's center resonant frequency to shift. By measuring the shift in the resonant frequency, the concentration of the molecules can be determined (Figure 8b). The resonance shift of CMUT after adsorbing gas molecules can be expressed as

(9) $$\Delta f=-\frac{f_{0}}{2m}\Delta m$$

The critical figure of merit of CMUT is mass sensitivity, which is defined as follows:

(10) $$S_{m}=\underset{\Delta m\to 0}{\lim}\frac{-1}{f}\frac{\Delta f}{\Delta m/A}=\frac{1}{2\rho t}$$

where Δ*m* is the loaded mass change, Δ*f* is the frequency shift, and *A* is the active sensor area.

In 2007, Park et al. published the first research paper on CMUT in the field of gas sensing.^[^ ^228^ ^]^ In their work, CMUT devices were coated with polymers such as polyallylamine hydrochloride, polyethylene glycol, and polyvinyl alcohol to detect various chemical substances. Experimental results demonstrated that CMUT exhibited a mass sensitivity of 1 fg Hz^−1^ and a volume sensitivity of 41.6 ppb Hz^−1^ toward water vapor. This work paved the way for CMUTs to be explored as VOC sensors.^[^ ^108^, ^233^, ^234^, ^235^, ^236^, ^237^, ^238^, ^239^, ^240^, ^241^ ^]^ Subsequently, Park et al. demonstrated a single‐channel CMUT sensor system capable of highly sensitive detection of DMMP.^[^ ^231^ ^]^ In this work, Park et al. functionalized CMUT with a PIB adsorbent film, targeting DMMP. Oscillator circuits were employed to track the resonance frequency of CMUT. Results showed that the CMUT chemical sensor achieved a detection limit for DMMP vapor of 56 ppb. However, the working frequency of this CMUT is relatively low, only 6 MHz. The lower resonant frequency limits the sensitivity of the CMUT. To address this issue, Lee et al. improved the structural design of the CMUT in another report, achieving a working frequency of 47.7 MHz.^[^ ^240^ ^]^ Additionally, Lee et al. utilized a poly(dimethylsiloxane) derivative as a functional coating, which provided high sensitivity to DMMP. Experimental results demonstrated that the sensor offered a sensitivity of 34.5 ppt Hz^−1^ and a detection limit as low as 10 ppb.

Although CMUT sensors exhibit advanced sensitivity in gas sensing, their high DC bias voltage hinders widespread application. For example, a CMUT operating at 50 MHz requires a 48 V DC bias.^[^ ^240^ ^]^ Currently, there are two main methods to reduce the required DC bias voltage for CMUTs: injecting charge into the dielectric layer and modifying the resonator sensor design.^[^ ^242^, ^243^ ^]^ Utilizing cavity‐floating islands for charge injection is an ingenious solution, offering long‐term charge storage (>1.5 years) without the need for additional bias voltage.^[^ ^244^ ^]^ However, this method requires more complex manufacturing processes and achieving reliable and repeatable charge injection across many units is challenging. Another approach is optimizing the size of the CMUT to reduce the DC voltage. For example, Park et al. reduced the DC voltage by increasing the resonator's diameter while maintaining a 50 nm thin vacuum gap height.^[^ ^137^ ^]^ The optimized CMUT sensing system operated at a voltage of 7 V with a resonant frequency of 6.7 MHz. Subsequently, four different polymers were coated on the CMUT surface to verify its sensing performance for VOCs. Experimental results showed that the low‐voltage CMUT sensor had a sensitivity of 1.5 Hz ppm^−1^ with a detection limit of 13 ppm. While increasing the CMUT diameter can reduce the DC operating voltage, it also sacrifices the sensitivity required for sensing. Therefore, balancing operating voltage and sensitivity remains a challenge that needs to be addressed in the future.

### 3.6 PMUT‐Based VOC Detection

**Figure 9:**
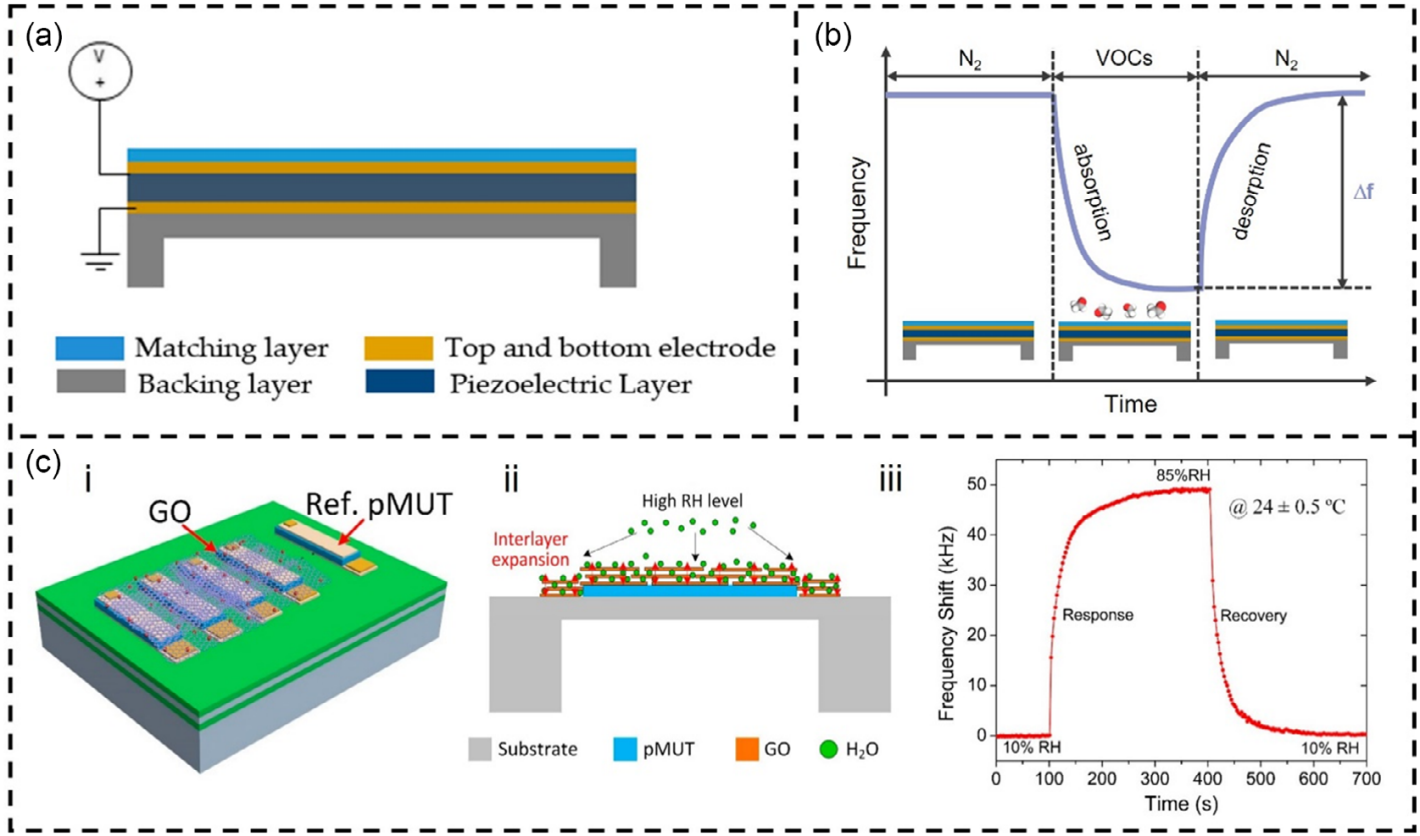
PMUT‐based VOC detection. a) The schematic diagram of PMUT. Reproduced with permission.^[^ ^248^ ^]^ Copyright 2018, MDPI. b) Schematic diagram of PMUT used as gas sensing principle. c) Functionalized PMUT array based on GO film for humidity sensing. i) Schematic structure of PMUT‐based humidity sensor. ii) Schematic illustration of humidity‐sensing mechanism of GO thin film coated PMUT. iii) Dynamic response of the PMUT humidity sensor. Reproduced with permission.^[^ ^136^ ^]^ Copyright 2018, MDPI.

PMUT is a sensor based on piezoelectric effect.^[^ ^245^, ^246^, ^247^ ^]^ Like CMUT, PMUT can also achieve analyte sensing through mass loading. In the previous section, we introduced the excellent sensing performance of chemical sensors based on CMUT for gas. However, CMUT also presents some issues, such as high bias voltage requirements and limitations in cavity structure. Sensors based on PMUT can effectively address these limitations. The ability to operate at lower voltages, flexibility in accommodating different sensing materials, and simple array configurations have made PMUT widely used in medical, environmental monitoring, and agricultural fields. In addition, PMUT gas sensors also have a large top electrode surface, providing sufficient space for the sensing material.^[^ ^248^ ^]^ The structure of PMUT includes a deflectable piezoelectric membrane sandwiched between top and bottom electrodes, clamped at the edges (**Figure** **9**a).^[^ ^248^ ^]^ When an alternating current voltage is applied to the top electrode, the piezoelectric layer vibrates at its resonance frequency due to the piezoelectric effect. The resonance frequency (*f*_0_) for a rectangular PMUT is expressed as follows:^[^ ^136^ ^]^

(11) $$f_{0}=\frac{0.494t}{W^{2}}\sqrt{\frac{E}{\rho \left(1-v^{2}\right)}\left[1+\frac{2}{3}{\left(\frac{W}{L}\right)}^{2}+{\left(\frac{W}{L}\right)}^{4}\right]}$$

where *t*, *L*, *W*, *E*, *ρ*, and *ν* are the thickness, length, width, Young's modulus, Poisson's ratio, and density of the rectangular membrane, respectively. In mass load sensing, the resonance shift of PMUT is the same as that of a cantilever beam.

When a PMUT gas sensor is exposed to the target gas, the sensing material on top of the PMUT absorbs the target gas molecules. The adsorption of these gas molecules causes changes in the effective mass and thickness of the sensing material. These changes, in turn, lead to a shift in the resonant frequency. By measuring the shift in resonant frequency, the concentration of the gas can be determined (Figure 9b). In 2018, Sun et al. proposed a linear PMUT array functionalized with GO for humidity sensing (Figure 9c).^[^ ^136^ ^]^ The hydrophilic groups in GO make it an excellent candidate for humidity detection. The study demonstrated that PMUT sensors exhibited outstanding humidity sensitivity (719 Hz per % RH) and relative sensitivity (271 ppm per % RH) within a wide sensing range of 10–90% RH. Additionally, the sensor featured fast response and recovery, minimal hysteresis, good stability, and linear frequency–temperature response. Subsequently, Sun et al. proposed a self‐powered multifunctional monitoring system integrating a hybrid integrated triboelectric nanogenerator (TENG) and piezoelectric microsensors.^[^ ^249^ ^]^ In this system, PMUTs were utilized as humidity sensors, and TENG was used to collect energy and power the sensor. The PMUT array was functionalized with PEI/GO composite material for detecting relative humidity. Results showed that the developed humidity sensor exhibited extremely high sensitivity (748 Hz per % RH) and relative sensitivity (290 ppm per % RH), rapid response and recovery (<22 s/53 s @ 90% RH), minimal hysteresis (3.07% RH), excellent linearity within a wide range of 20–90% RH, and good stability. However, according to current literature, PMUT sensors are primarily used for medical imaging and fingerprint applications, rather than gas sensing. Exploring the application of PMUTs in gas sensing could be an interesting area of research.

## 4 Nano‐Optical Sensors for VOC Detection

### 4.1 Infrared Plasmonic Nanoantenna‐Based VOC Detection

**Figure 10:**
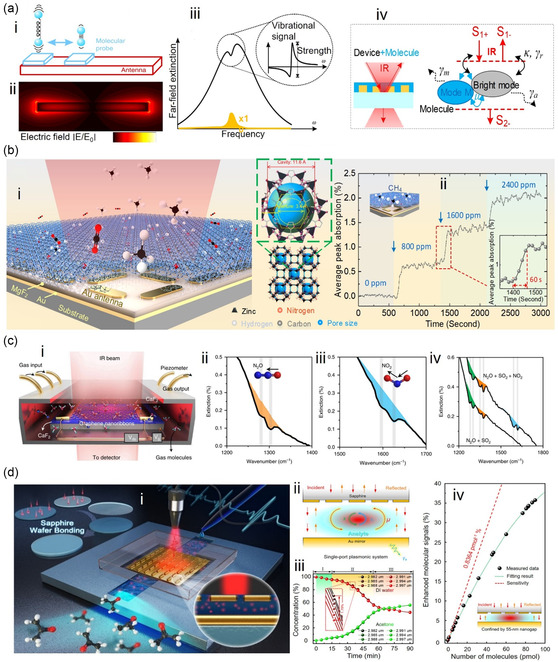
Plasmonic nanoantenna‐based VOC detection in the IR region. a) Sensing principle. i) Molecules are coupled with the local near‐field of the nanoantenna. ii) Simulated local near‐field distribution. iii) The vibrational modes of molecules are enhanced by the local near‐field. Reproduced with permission.^[^ ^262^ ^]^ Copyright 2013, Springer Nature. iv) Coupled mode model of nanoantennas for theoretical analysis. Reproduced with permission.^[^ ^129^ ^]^ Copyright 2022, Springer Nature. b) Metal nanoantennas integrating gas enrichment for gas detection. i) Schematic representation of the gas sensing platform. ii) Dynamic sensing behaviors of the platform. Reproduced with permission.^[^ ^270^ ^]^ Copyright 2020, John Wiley and Sons. c) Graphene plasmons for VOC gas identification. i) Schematic representation. ii–iv) Identification of different nitrogen oxides. Reproduced with permission.^[^ ^274^ ^]^ Copyright 2019, Springer Nature. d) Nanofluidic nanoantenna‐based platform for the detection of VOCs in the liquid phase. i) Schematic representation. ii) Coupling principle for analytes and nanoantennas. iii) In situ dynamic monitoring for molecular diffusion in DI water. iv) Experimentally molecular signal enhancement versus the number of acetone molecules. Reproduced with permission.^[^ ^275^ ^]^ Copyright 2020, American Chemical Society.

The IR plasmonic nanoantenna is a nanoscale artificially structured material that can manipulate light at ultrahigh levels by exciting localized surface plasmons, providing an ultrastrong near‐field.^[^ ^250^, ^251^, ^252^, ^253^, ^254^, ^255^, ^256^, ^257^, ^258^, ^259^, ^260^, ^261^ ^]^ For example, the nanorods with a near‐field distribution of lightning rods can provide nearly 1000‐fold field enhancement (**Figure** **10**a(i,ii)).^[^ ^262^ ^]^ By trapping VOC molecules in the near‐field of the nanoantenna, the IR absorption of VOCs can be enhanced through SEIRA.^[^ ^38^, ^263^, ^264^, ^265^, ^266^, ^267^, ^268^ ^]^ In this process, light interacts with the coupling system of the nanoantenna and molecule, instead of interacting directly with the VOC. The SEIRA effect can address the issue of low IR absorption cross section of VOC molecules. In Figure 10a(iii), the spectrum of the nanoantenna demonstrates that weak molecular vibration signals are significantly amplified due to the near‐field enhancement of the nanoantenna. The enhancement process can be analyzed by temporal coupled‐mode theory (TCMT). Figure 10a(iv) illustrates the general model based on TCMT,^[^ ^129^ ^]^ consisting of a single cavity coupled with inward and outward traveling waves. The coupled‐mode equations can be written as

(12) $$\frac{dA}{dt}=j\omega_{0}A-\left(\gamma_{r}+\gamma_{a}\right)A+j\mu M+\kappa S_{1+}$$

(13) $$\frac{dM}{dt}=j\omega_{m}M-\gamma_{m}M+j\mu A$$

where the bright mode (*A*) of the nanoantenna is coupled with the incident light (*S*_1+_), and the coupling coefficient is *κ*. It experiences damping at a rate *γ*_r_ and *γ*_a_, and the molecular damping *γ*_m_ is coupled to the nanoantennas via a coupling strength *μ*. The lossy molecular vibration (denoted as *M*) is coupled (coefficient *μ*) to the bright‐mode nanoantenna as a purely dissipative mode. Through calculating the differential spectra of the nanoantennas with/without coupled molecules at resonance without detuning, the enhancement of molecular signal can be expressed as

(14) $$\Delta R_{m}=\frac{2\mu^{2}{\left(\gamma_{r}/\gamma_{a}\right)}^{2}}{\gamma_{a}\gamma_{m}{\left(1+\gamma_{r}/\gamma_{a}\right)}^{3}}$$

According to the previously reported literature, the signal enhancement factor can be up to 10^7^.^[^ ^269^ ^]^ The above equation highlights that in order to achieve greater signal enhancement, it is imperative to enhance the coupling coefficient (*μ*) between the molecule and the near field.

For gas detection, a common way to improve *μ* is the use of gas‐selective‐trapping materials. Zhou and collaborators integrated MOFs with multiresonant nanoantennas for the ultrasensitive detection of multiple gases (Figure 10b).^[^ ^270^ ^]^ The integration of MOFs with nanoantennas requires careful consideration of several factors. First, the absorption peak of the gas‐selective‐trapping material should not overlap with the absorption peak of the target gas molecule. Second, the resonance frequency of the antenna should match the molecular absorption peak to maximize enhancement. Notably, in the antenna‐molecule coupling system, the maximum enhancement is not always obtained when the molecular vibration and resonance are perfectly matched. The loss engineering of the antenna plays a crucial role in improving the performance of the sensing system, particularly by matching the loss of the antenna and free space. Furthermore, multiresonant antenna designs can enhance the sensing capability for detecting multiple gases, as the vibrations of different target gases are often located in distinct frequency ranges. Multiresonance can be achieved through the combination of antennas with different lengths. Moreover, sensing platforms that rely on chemical mechanisms for gas enrichment have demonstrated faster response times compared to sensors based on physical adsorption mechanisms. This is an important consideration in the development of high‐performance VOC gas sensing systems. Overall, the implementation of multiresonant antennas and gas‐enrichment materials can play important roles in the design of efficient and effective VOC gas sensing platforms.

Antennas that utilize2D materials are increasingly being explored for the detection of VOC gases. This is due to the unique properties of 2D materials, such as their large specific surface area and excellent electrical conductivity, which make them suitable for use as both VOC gas‐enrichment materials and nanoantennas.^[^ ^271^, ^272^, ^273^ ^]^ Specifically, in the case of 2D material‐based nanoantennas, the coupling of VOC molecules to the near‐field of the antenna occurs within the 2D material itself, resulting in improved sensitivity (*μ*). As a result, the integration of 2D materials into the design of nanoantennas offers promising opportunities for the development of highly sensitive and selective gas sensing platforms. For example, in 2019, Hu and collaborators developed graphene nanoribbons to excite surface plasmons to detect the vibrations of multi‐VOC gases (Figure 10c).^[^ ^274^ ^]^ The mechanism of gas adsorption in this study involved the adsorption and accumulation of gas molecules on the graphene layer through image attraction force and defect adsorption. The detected signal corresponded to a layer of gas molecules adsorbed on the graphene surface at a concentration of 800 zeptomole per μm^2^. The study successfully detected several gas types, including SO_2_, NO_2_, N_2_O, and NO, with a gas response time of 1 min. Future research efforts should aim to improve the crystal quality and mobility of graphene, which would enhance the quality factor and field enhancement effect of graphene plasmons.

For the detection of liquid VOCs, an efficient approach is to incorporate microfluidic technology with plasmonic antennas. Nanoantennas can be engineered within microfluidic channels, enabling the antennas to directly interact with the analytes present within the channel. As demonstrated by Xu et al. in 2020 (Figure 10d(i)),^[^ ^275^ ^]^ the dielectric layer of the metal–insulator–metal absorber was substituted with nanofluidic channels by employing a low‐temperature interfacial heterosapphire wafer direct bonding technique. Therefore, the analyte in the microfluidic channel and the near‐field has an excellent spatial overlap between the antenna and the metal reflector. The resulting platform demonstrated exceptional resolution (0.29%) and ultrahigh sensitivity (0.8364 pmol/1%). Moreover, the platform allowed for in situ real‐time dynamic monitoring of the diffusion of acetone molecules in deionized water. Overall, the plasmonic antenna is a powerful VOC detection platform, which can realize VOC detection and identification in gaseous and liquid states.

### 4.2 All‐Dielectric Metamaterial‐Based VOC Detection

**Figure 11:**
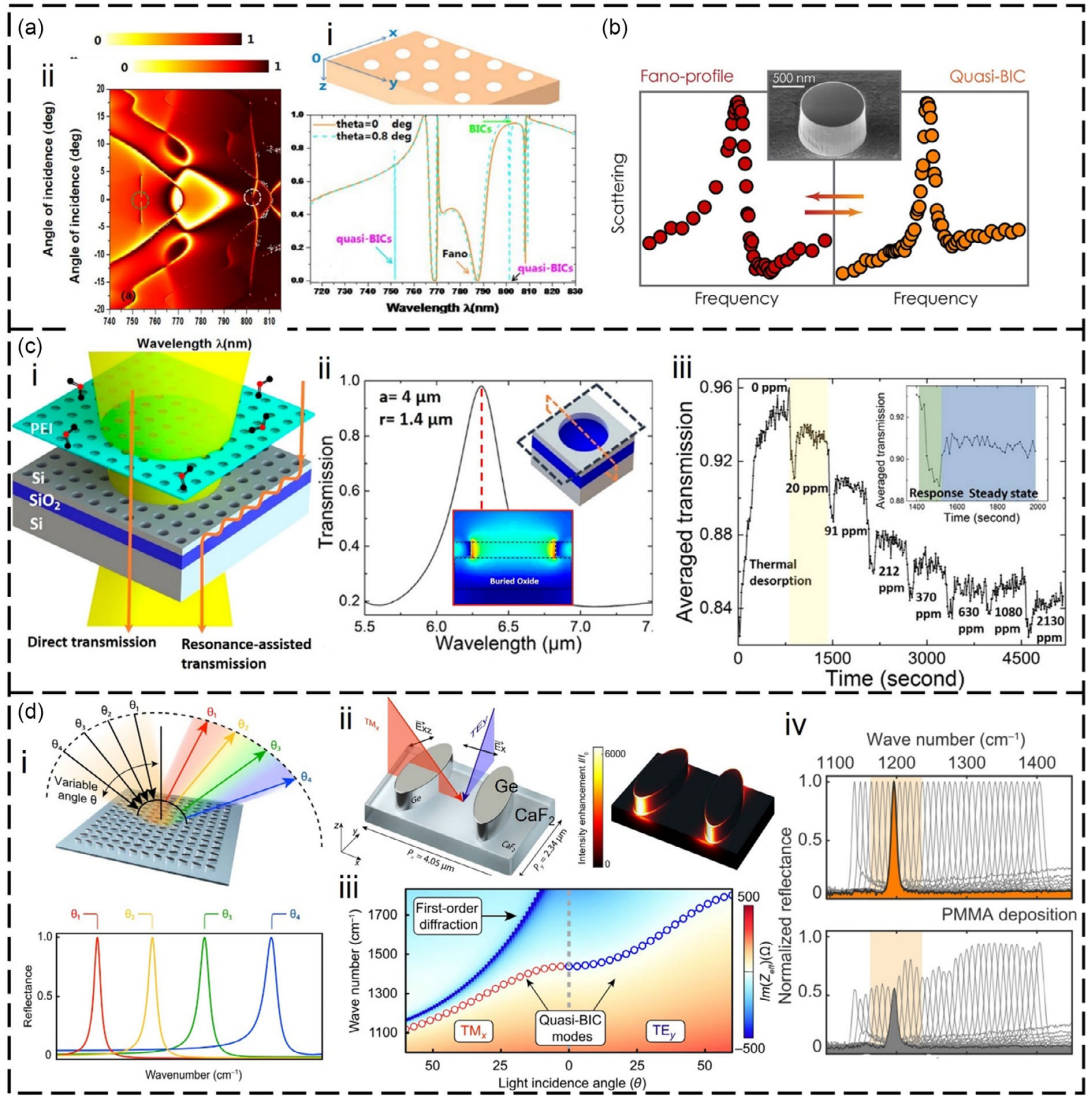
All‐dielectric metamaterial‐based VOC detection in the IR region. a) Fano and quasi‐BIC modes based on all‐dielectric metamaterials. i) Device diagram. ii) Corresponding transmission spectrum as functions of the wavelength and the incidence angle. iii) Transmission coefficient at 0° and 0.8° incidence angles showing the appearance of quasi‐BICs and Fano resonances. Reproduced with permission.^[^ ^286^ ^]^ Copyright 2019, IOP Publishing. b) Mode transition from quasi‐BICs to Fano resonances. Reproduced with permission.^[^ ^287^ ^]^ Copyright 2021, American Chemical Society. c) All‐dielectric resonators for gas sensing. i) Schematic diagram. ii) Simulated transmission spectrum. iii) Dynamical behavior of gas sensing demonstration. Reproduced with permission.^[^ ^127^ ^]^ Copyright 2018, American Chemical Society. d) Angle‐multiplexed quasi‐BIC‐based metamaterials for molecule detection. i) Schematic diagram of the device with specific resonance frequency for every incidence angle. ii) The device consists of asymmetric ellipse‐shaped antennas. iii) Effective reactance lm(*Z*_eff_) of the quasi‐BIC as a function of the incident angle showing different excitation modes. iv: Retrieval of vibrational fingerprint information using the device. Reproduced with permission.^[^ ^288^ ^]^ Copyright 2019, American Association for the Advancement of Science.

Metamaterials are artificial periodic structures that can manipulate the flow of light in a variety of ways.^[^ ^276^, ^277^, ^278^, ^279^, ^280^, ^281^, ^282^ ^]^ In the IR region, the vibration of the molecule is coupled with the all‐dielectric Mie resonance and then enhanced through the SEIRA effect.^[^ ^283^, ^284^, ^285^ ^]^ The choice of all‐dielectric resonators is crucial for practical applications. Fano resonance and bound state in the continuum (BIC) are two modes suitable for SEIRA (**Figure** **11**a).^[^ ^286^ ^]^ BIC is a type of wave phenomenon where a state can be perfectly confined or localized within a specific area, despite the presence of an open and continuous background, which normally would allow the wave to spread out. From a spectral perspective, Fano resonance is broad, while BIC has a narrow band with an ultrahigh *Q* value. The two modes can be converted by adjusting the structural parameters of the antenna, as demonstrated in Figure 11b.^[^ ^287^ ^]^ The VOC detection based on Fano resonance is analogous to the plasmonic VOC detection previously discussed. However, the low loss of dielectric materials results in a higher Q‐factor and weaker heating effects compared to metallic materials, resulting in higher detection sensitivity. In 2020, Chang et al. developed an all‐dielectric SEIRA‐based photonic crystal platform that integrates gas‐selective‐trapping films for highly sensitive gas detection, as shown in Figure 11c.^[^ ^127^ ^]^ The platform's enhancement of gas molecular vibrations is the result of two mechanisms induced by guided resonances, namely, an enlarged effective path length due to multiple round trips within the plate and a uniformly enhanced electric field along the sidewall. The platform achieved a detection limit of 20 ppm CO_2_ with a response time of 2 min. This detection scheme can be easily extended to detect VOC by changing it to a VOC‐selective‐trapping material, such as polyaniline, 2D materials, and so on.

BIC‐based sensors exhibit distinct molecular vibration detection capabilities compared to those based on Fano resonance. Due to the narrow bandwidth and high Q‐factor, BIC devices require a pixelated design to cover a wide range of molecular vibrations, as shown in Figure 11d(i).^[^ ^288^ ^]^ The pixelated design can be controlled by changing the dimensions of the nanoantenna or by adjusting the incident angle of the mid‐IR light. This approach of angle‐multiplexing allows for an extensive range of on‐demand resonances in the mid‐IR from a solitary nanoantenna chip, limited only by the range of light incidence angles (Figure 11d(iii)). At angular positions where the resonance frequency of the BIC nanoantenna coincides with the vibrational mode of the analyte molecule, a strong modulation of the far‐field optical response occurs due to the enhanced accessibility of the surface‐enhanced electrical near‐field (Figure 11d(ii)). By retrieving angle‐resolved reflection signals from such BIC nanoantennas before and after the adsorption of analyte molecules, it is possible to obtain the complete spectral information of the molecular absorption fingerprints (Figure 11d(iv)). The presented biomolecule detection scheme has significant implications for the detection of VOCs. By designing a 2D pixelated array, a BIC sensor with ultrahigh Q‐factor can be transformed into a spatial absorption map that resembles a barcode. This approach enables imaging without the need for spectral analysis, frequency scanning, or moving mechanical parts.

### 4.3 SERS‐Based VOC Detection

**Figure 12:**
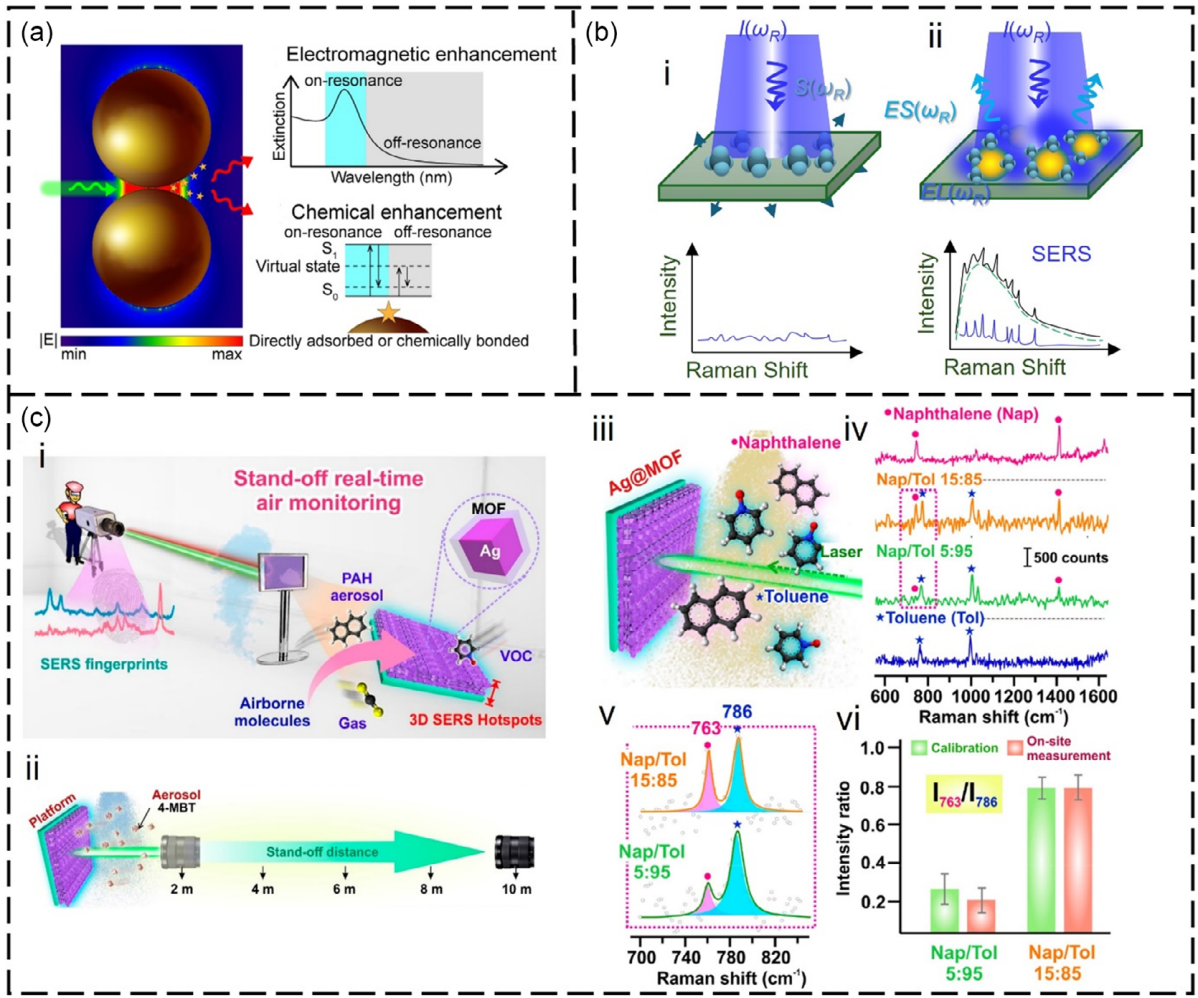
SERS‐based VOC detection in the visible region. a) Scheme of the SERS effect. Reproduced with permission.^[^ ^293^ ^]^ Copyright 2021, AIP Publishing. b) Schematic diagrams of signal enhancement of SERS. Reproduced with permission.^[^ ^301^ ^]^ Copyright 2023, The Royal Society of Chemistry. c) Demonstration of SERS‐based VOC gas detection. i) Concept of the stand‐off SERS platform. ii) Scheme showing the stand‐off VOC gas detection using the SERS platform. iii) Demonstration of the stand‐off detection of aerosolized toluene and naphthalene. iv) Obtained stand‐off multiplex spectra. v) Spectral analysis of characteristic signals of the VOCs. vi) Comparison of the signal intensity ratio of the platform between calibration spectra and outdoor spectra. Reproduced with permission.^[^ ^125^ ^]^ Copyright 2019, American Chemical Society.

SERS is a powerful analytical technique that exploits the enhanced electromagnetic field generated by metallic nanoparticles to amplify the Raman scattering signal of VOC molecules.^[^ ^42^, ^289^, ^290^, ^291^, ^292^ ^]^ The enhancement mechanism of SERS can be broadly classified into electromagnetic enhancement and chemical enhancement (**Figure** **12**a).^[^ ^293^ ^]^ Electromagnetic enhancement is the dominant mechanism in SERS, and it arises from the interaction between the localized electromagnetic field generated by metallic nanoparticles and the analyte molecule adsorbed on their surface. The electromagnetic enhancement includes the following effects. The first is localized surface plasmon resonance (LSPR).^[^ ^294^, ^295^ ^]^ When metallic nanoparticles are irradiated with light, they can undergo a collective oscillation of the conduction electrons on their surface. This LSPR can generate a strong electromagnetic field in the vicinity of the nanoparticle, which can significantly enhance the Raman scattering signal of the adsorbed molecule. The second is hotspots.^[^ ^296^ ^]^ Hotspots are regions on the metal surface where the electromagnetic field is highly concentrated and amplified. Hotspots can arise due to the geometrical features of the nanoparticles or the interaction between adjacent nanoparticles, resulting in an enhancement of the Raman scattering signal. The third is surface roughness.^[^ ^297^ ^]^ The surface roughness of the metal nanoparticle can also contribute to the electromagnetic enhancement of SERS. The roughness can create additional hotspots and enhance the electromagnetic field, leading to further amplification of the Raman scattering signal. In terms of chemical enhancement, it arises from the modification of the electronic structure of the analyte molecule due to its interaction with the metal surface.^[^ ^298^ ^]^ The chemical enhancement includes charge transfer^[^ ^299^ ^]^ and resonance Raman scattering.^[^ ^300^ ^]^ Charge transfer between the metal surface and the adsorbed molecule can lead to the formation of a surface complex with an altered electronic structure. The complex can exhibit enhanced Raman scattering due to the modification of its vibrational modes. Resonance Raman scattering occurs when the excitation energy of the incident photon is in resonance with the electronic transitions of the analyte molecule. In SERS, the excitation energy can be tuned to match the plasmon resonance of the metal nanoparticle, resulting in a resonance enhancement of the Raman scattering signal. Based on the above enhancement mechanism, the Raman signal can be greatly amplified (Figure 12b).^[^ ^301^ ^]^

Gas‐selective‐capturing materials are important for SERS‐based VOC gas detection because of the following advantages. The first is enhancing the concentration of the target gas molecules and improving the signal‐to‐noise ratio of the SERS measurement.^[^ ^302^, ^303^ ^]^ The gas molecules adsorbed on the capturing materials can be concentrated in a small volume, leading to a higher probability of interaction with the SERS‐active metal nanoparticles. Moreover, the capturing materials can provide a stable environment for the SERS measurement, preventing the target gas molecules from diffusing away from the detection region. The second is improving the selectivity of the detection system.^[^ ^304^, ^305^, ^306^ ^]^ The gas‐selective‐capturing materials can be designed to have specific binding sites that can selectively recognize the target gas molecules based on their chemical properties, such as size, shape, and functional groups. The gas‐selective‐capturing materials can be in the form of porous materials, such as zeolites, MOFs, and porous polymers, or surface‐functionalized metal nanoparticles.^[^ ^307^, ^308^, ^309^ ^]^ These materials have high surface areas and specific binding sites that can selectively adsorb the target gas molecules. For instance, Phan‐Quang and co‐workers demonstrated a MOF‐integrated SERS platform for stand‐off and real‐time VOC gas detection (Figure 12c).^[^ ^125^ ^]^ In this platform, MOFs are used to adsorb and concentrate polycyclic aromatic hydrocarbon mixtures into the near‐field of the SERS substrate. Ag nanocubes were used as the SERS substrate. A stand‐off Raman system with a large collection volume of 1 × 1 ×3.9 mm was utilized to acquire the SERS signal (Figure 12c(i,ii)). Experimental results demonstrate that this method can remotely measure the concentration of VOC molecules down to tens of ppm level. In addition, the system successfully detected the VOC mixture of naphthalene (Nap) and toluene (Tol) with concentrations ranging from Nap/Tol 850:3500 ppm to Nap/Tol 49:3500 ppm (Figure 12c(iii,vi)).

### 4.4 SPR/LSPR‐Based VOC Detection

**Figure 13:**
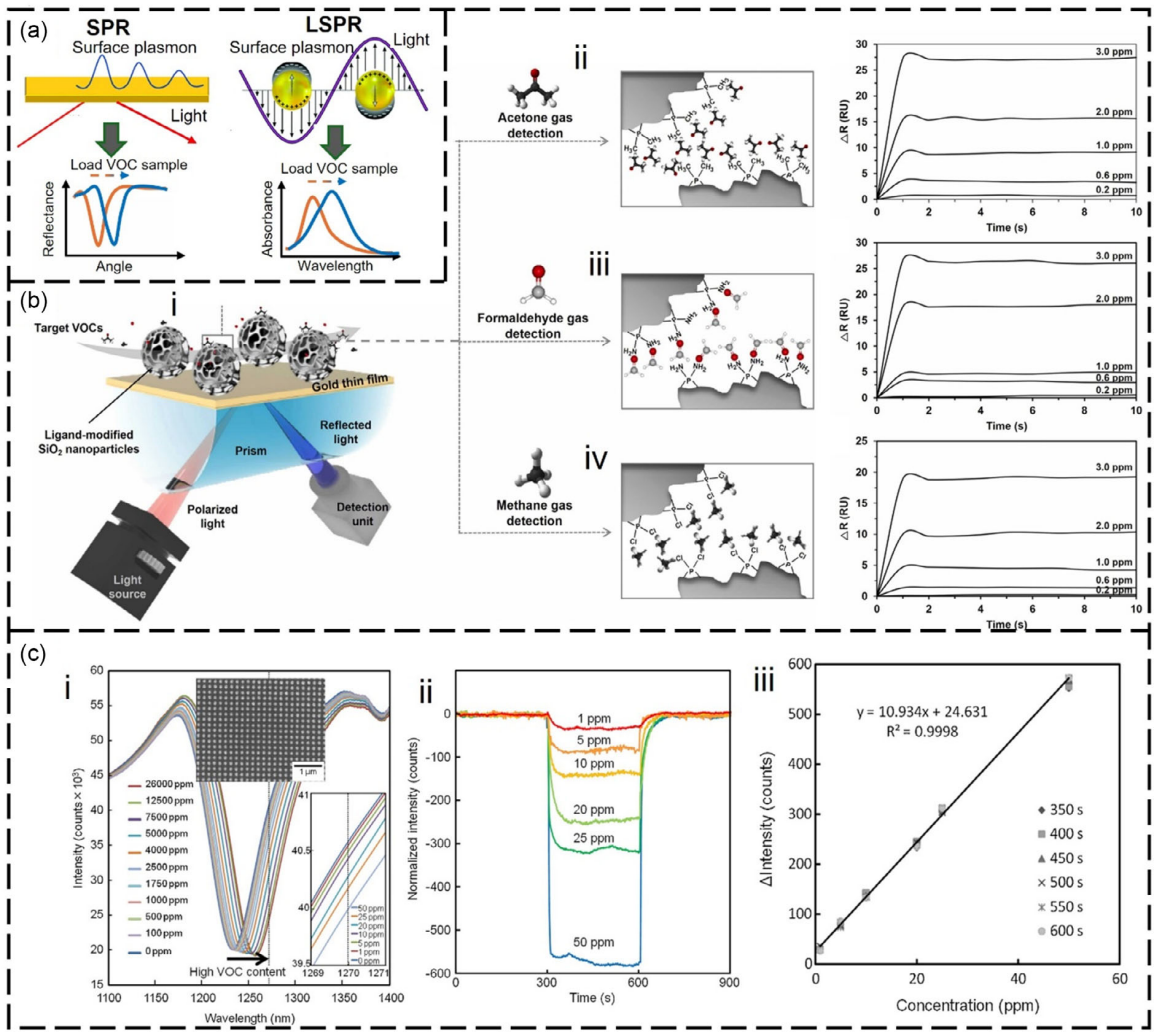
SPR/LSPR‐based VOC detection. a) Schematic diagrams of SPR and LSPR for VOC detection. Reproduced with permission.^[^ ^310^ ^]^ Copyright 2020, Royal Society of Chemistry. b) SPR‐based detection of multiple VOCs. i) Schematic diagram of the platform. ii) Acetone gas detection. iii) formaldehyde gas detection. iv) methane gas detection. Reproduced with permission.^[^ ^124^ ^]^ Copyright 2022, Elsevier. c) LSPR‐based VOC detection. i) The LSPR spectrum changes when exposed to different concentrations of toluene. Inset is the SEM image. ii) The dynamic response in the presence of toluene of 1–50 ppm. iii) The calibration curves obtained from the dynamic response. Reproduced with permission.^[^ ^320^ ^]^ Copyright 2014, Elsevier.

In addition to probing VOC molecules by coupling with their vibrational modes, as demonstrated in **Figure** **13**, the plasmon can also interact with their refractive properties to achieve detection. Figure 13a shows the VOC detection mechanism using SPR and LSPR.^[^ ^310^ ^]^ SPR is a label‐free optical technique used to detect gas molecules based on changes in the RI of a gas‐sensitive layer coated on a metal film.^[^ ^311^, ^312^, ^313^, ^314^ ^]^ The principle of SPR gas sensing involves measuring the shift in the SPR angle caused by the interaction between the gas molecules and the gas‐sensitive layer. In SPR gas sensing, a thin metal film, typically gold or silver, is coated with a gas‐sensitive layer, such as a polymer or metal‐oxide film.^[^ ^311^ ^]^ When a gas molecule interacts with the gas‐sensitive layer, it causes a change in the RI of the layer, which, in turn, alters the SPR response of the metal film. The amount of RI change depends on the concentration and nature of the gas molecule. The SPR angle is the angle of incidence at which the surface plasmon resonance occurs, resulting in a dip in the reflected light intensity. The SPR angle is dependent on the RI of the metal film and the dielectric constant of the medium above the film, which can be modified by the adsorption of gas molecules on the gas‐sensitive layer. When gas molecules are adsorbed on the gas‐sensitive layer, the RI of the layer changes, resulting in a shift in the SPR angle. For instance, Choi and co‐workers demonstrated a Kretchmann configuration SPR with mesoporous SiO_2_ nanomaterials as gas‐selective‐capturing materials for VOC detection, including acetone, formaldehyde, and methane gases (Figure 13b).^[^ ^124^ ^]^ To enhance both sensitivity and gas selectivity, SiO_2_/Au nanocomposites are coated with a Cl‐functionalized SAM to provide numerous acceptor sites, allowing VOC gas to enter the acceptor sites in the pores with ease. Moreover, the utilization of nanocomposites also facilitates the extension of the evanescent field, alongside a dielectric spacing layer that effectively amplifies the SPR signal. Experimental results demonstrated that this method exhibits linearity and response times of less than 2 s for gas concentrations within the 0.2–3.0 ppm range. The LODs of this method for acetone, formaldehyde, and methane gas reached 0.3, 0.6, and 0.5 ppm, respectively.

LSPR is based on the resonant excitation of surface plasmons in small metal nanoparticles or nanoantennas (Figure 13a). The plasmons in these nanoparticles are localized and are excited by the interaction of light with the metal nanoantennas.^[^ ^315^ ^]^ The LSPR response of the nanoantennas is highly sensitive to the local environment, such as the RI and the presence of analytes. This makes LSPR an excellent tool for detecting VOC gases in a label‐free manner.^[^ ^316^, ^317^, ^318^, ^319^ ^]^ The LSPR frequency is the frequency of the resonant oscillations of the conduction electrons in the metal nanoparticles. The LSPR frequency is dependent on the size, shape, and composition of the nanoparticles, and can be modified by the adsorption of gas molecules on the gas‐sensitive layer. For instance, Takimoto and his team successfully developed an LSPR sensing platform using chips patterned with Au nanoparticles and coated with porous silica.^[^ ^317^ ^]^ Using the sol–gel method, the nanoantennas were coated with porous silica, allowing for the detection of 20 ppm of SO_2_. The sensitivity of the chips coated with porous silica was found to be four times higher than that of the uncoated chips. The sensitivity to SO_2_ gas was significantly improved by applying chemical vapor deposition to modify the porous silica layer with 3‐aminopropyltrimethoxysilane. This modification resulted in a 13‐fold increase in sensitivity compared to the unmodified porous‐silica‐coated chip. In 2014, Monkawa et al. developed a highly sensitive and wide‐range LSPR sensor by patterning 400 nm diameter, 800 nm wide Au dots on a quartz glass substrate (Figure 13c).^[^ ^320^ ^]^ The LSPR sensor exhibits a peak at 1230 nm in the near isolated area, allowing for the detection of RI changes induced by VOC. Additionally, they demonstrated that coating the Au‐dot pattern with a thin porous silica film enhances the adsorption of VOCs, further increasing the RI change. The LSPR sensor enables the real‐time detection of toluene vapors with concentrations ranging from 1 to 26 000 ppm, with a calculated limit of detection of 0.4 ppm.

### 4.5 Waveguide‐Based VOC Detection

**Figure 14:**
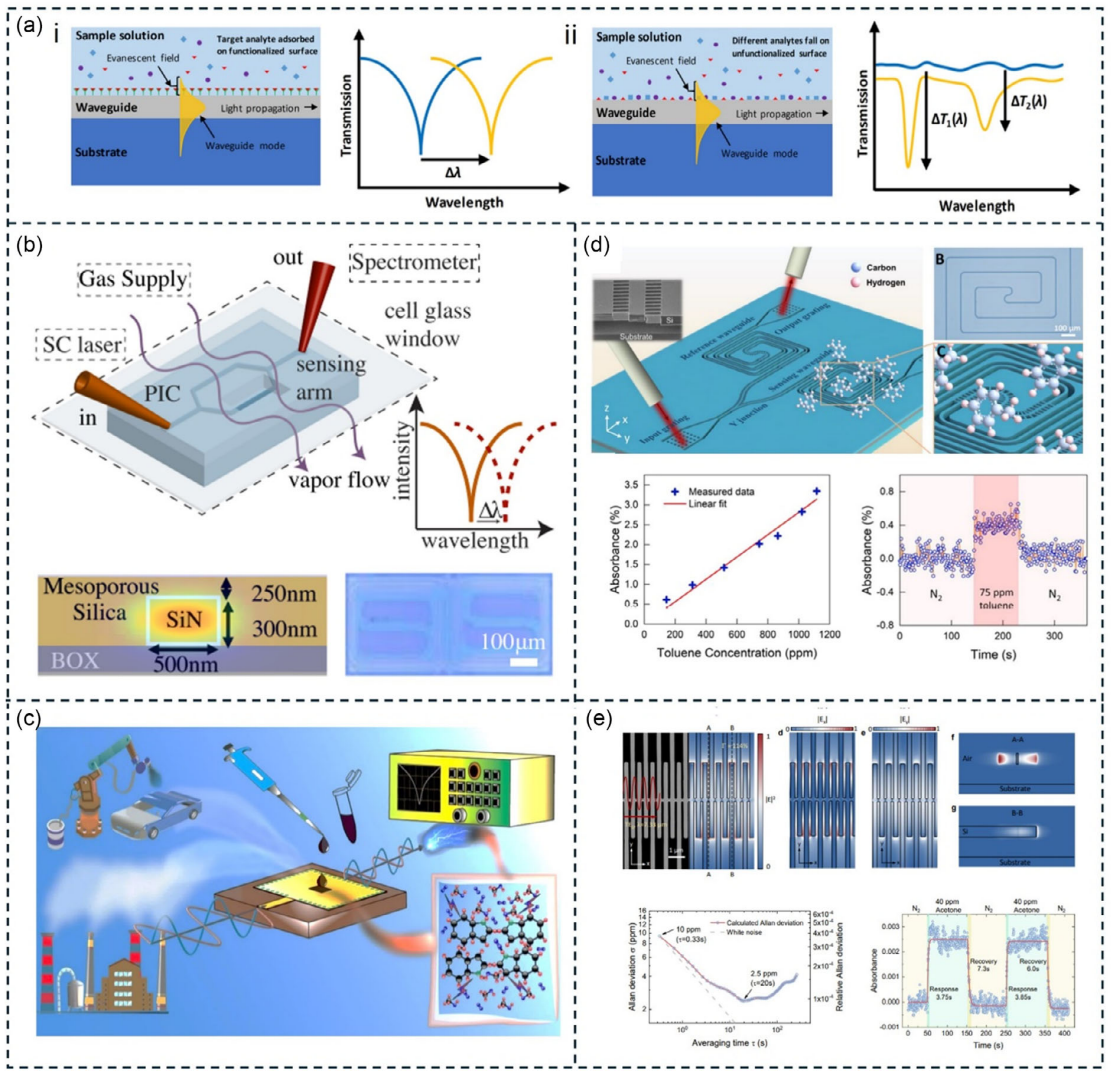
Waveguide‐based VOCs detection. a) i) RI‐based sensor with its surface functionalized to selectively adsorb analyte. ii) Absorption‐based sensor without surface functionalization. Reproduced with permission.^[^ ^325^ ^]^ Copyright 2020, Springer Nature. b) RI gas sensor with functionalized silicon nitride photonic circuits. Reproduced with permission.^[^ ^330^ ^]^ Copyright 2020, AIP Publishing. c) Methanol gas using a polyindole‐embedded substrate integrated waveguide microwave sensor. Reproduced with permission.^[^ ^332^ ^]^ Copyright 2020, American Chemical Society. d) Suspended silicon waveguide platform with subwavelength grating metamaterial cladding for toluene vapor detection. Reproduced with permission.^[^ ^256^ ^]^ Copyright 2021, De Gruyter. e) Metamaterial‐assisted comb waveguide for ultrasensitive long‐wave IR gas spectroscopy. Reproduced with permission.^[^ ^128^ ^]^ Copyright 2022, American Chemical Society.

Waveguide‐based sensors offer an attractive solution for molecular detection.^[^ ^321^, ^322^, ^323^, ^324^ ^]^ These sensors typically consist of a waveguide layer with a high RI. This waveguide layer allows light or electromagnetic waves to propagate within it while confining their propagation in space. Most waveguide sensors operate based on evanescent field sensing, where sensitivity is directly related to the size of the guided mode's evanescent field. Generally, there are two main sensing schemes for waveguide sensors: RI sensing and absorption sensing.^[^ ^325^ ^]^ RI sensors detect the real part (*n*) of the RI of the medium (**Figure** **14**a(i)). The resonant frequency of waveguide sensors varies with changes in the surface RI.^[^ ^326^ ^]^ RI sensors are suitable for small volume samples. One advantage of RI sensors is that small changes in n over short distances can result in significant changes in the phase of the propagating wave, achieving very high sensitivity through different optical devices. Mach–Zehnder interferometers (MZIs) and microresonators are the most widely used optical devices in RI sensing.^[^ ^327^, ^328^, ^329^ ^]^ For instance, Antonacci et al. demonstrated a photonic chip based on silicon nitride waveguides for achieving highly sensitive VOCs sensing (Figure 14b).^[^ ^330^ ^]^ By monitoring the output spectral pattern of an integrated unbalanced MZI, low‐concentration chemical vapors were detected. To enhance the sensitivity of the instrument, the sensing arm of the MZI was coated with a functionalized mesoporous silica layer that preferentially adsorbs organic solvents. When gas vapors are absorbed by the top cladding mesoporous layer, light propagating along the exposed waveguide experiences different refractive indices, resulting in wavelength shifts of the interference fringes generated by the MZI. Experimental results showed that this waveguide sensor could detect various VOC vapors such as acetone, isopropanol, and ethanol, with detection limits of 65, 247, and 1.6 ppb, respectively. However, RI sensors lack selectivity. In other words, RI sensors cannot determine the substances causing changes in RI. Additionally, in the case of multicomponent VOCs, RI sensors cannot attribute RI changes to specific VOC. Surface functionalization is an effective solution to improve the poor selectivity of RI sensors.^[^ ^331^ ^]^ To enhance specificity, Kumar et al. deposited polyindole (PIn) on the surface of an integrated waveguide to achieve quantitative and qualitative sensing of methanol gas (Figure 14c).^[^ ^332^ ^]^ PIn exhibits high dielectric performance and strong hydrogen bonding, resulting in high sensitivity to methanol gas. Interaction between methanol gas and PIn leads to changes in Pin's high‐frequency conductivity and dielectric properties, causing resonance frequency shifts in the waveguide. Concentration measurement of methanol gas is achieved by tracking the resonance frequency shift. The study demonstrated that the sensitivity of this waveguide sensor was 3.3 kHz ppm^−1^. Additionally, the sensor exhibited excellent stability and repeatability, with required response and recovery times of 95 and 120 s, respectively.

Another sensing scheme for waveguide sensors is absorption sensing.^[^ ^333^, ^334^, ^335^ ^]^ Absorption sensing detects the imaginary part (*k*) of the RI of the medium (Figure 14a(ii)). Unlike detecting the real part of the RI, detecting the imaginary part of the RI can be selective. The imaginary part of the RI describes the medium's ability to absorb light. This absorption ability resulting from the imaginary part is related to the frequency at which the medium absorbs light, which influences the shape and intensity of the absorption spectrum. When light passes through the medium, some of the light's energy is absorbed by the medium and converted into other forms of energy, such as heat. This absorption occurs because molecules in the medium absorb the energy of light, causing molecular vibrations and rotations. The energy required for these vibrations and rotations is closely related to the frequency of light. Different media absorb light of different frequencies to varying degrees, forming absorption spectra. Therefore, we can specify the presence of different substances in the sample through absorption spectra. Consequently, the absorption sensing scheme does not require surface functionalization of the waveguide. However, the sensitivity of this technique is limited due to the short interaction length between light and the sample. To overcome this issue, Liu et al. proposed a suspended silicon waveguide platform for long‐wave IR gas sensing, utilizing subwavelength grating (SWG) metamaterial cladding (Figure 14d).^[^ ^256^ ^]^ The platform offers high sensitivity and rapid response. It operates in the 6.4–6.8 μm wavelength range, with a waveguide propagation loss of 3.9 dB cm^−1^. Using toluene vapor as an example, the platform demonstrates a detection limit of 75 ppm, a response time of 0.8 s, and a recovery time of 3.4 s. The platform requires no additional adsorption coatings and is suitable for environmental monitoring and clinical diagnostics. Further optimization could achieve even lower detection limits, showcasing its significant potential for various real‐time applications. Subsequently, Liu et al. used all‐dielectric metamaterials to confine more of the evanescent fields in the air, thereby improving sensing sensitivity (Figure 14e).^[^ ^128^ ^]^ The extraordinary optical field confinement is enabled by large longitudinal electric field discontinuity at periodic high‐index‐contrast Si/air interfaces in SWG metamaterial, together with its unique features in RI engineering. The waveguide operates in the long‐wave IR (6–14 μm) spectrum, which contains absorption fingerprints of many chemical bonds. By optimizing the period and duty cycle of the waveguide, an external confinement factor of 113% and low propagation loss of 4.7 dB cm^−1^ were achieved. Finally, Liu et al. validated the sensing performance of the waveguide using acetone vapor, demonstrating a detection limit of 2.5 ppm and fast dynamic response (response time ≈3.8 s, recovery time ≈6.6 s).

### 4.6 THz and Microwave VOC Detection

**Figure 15:**
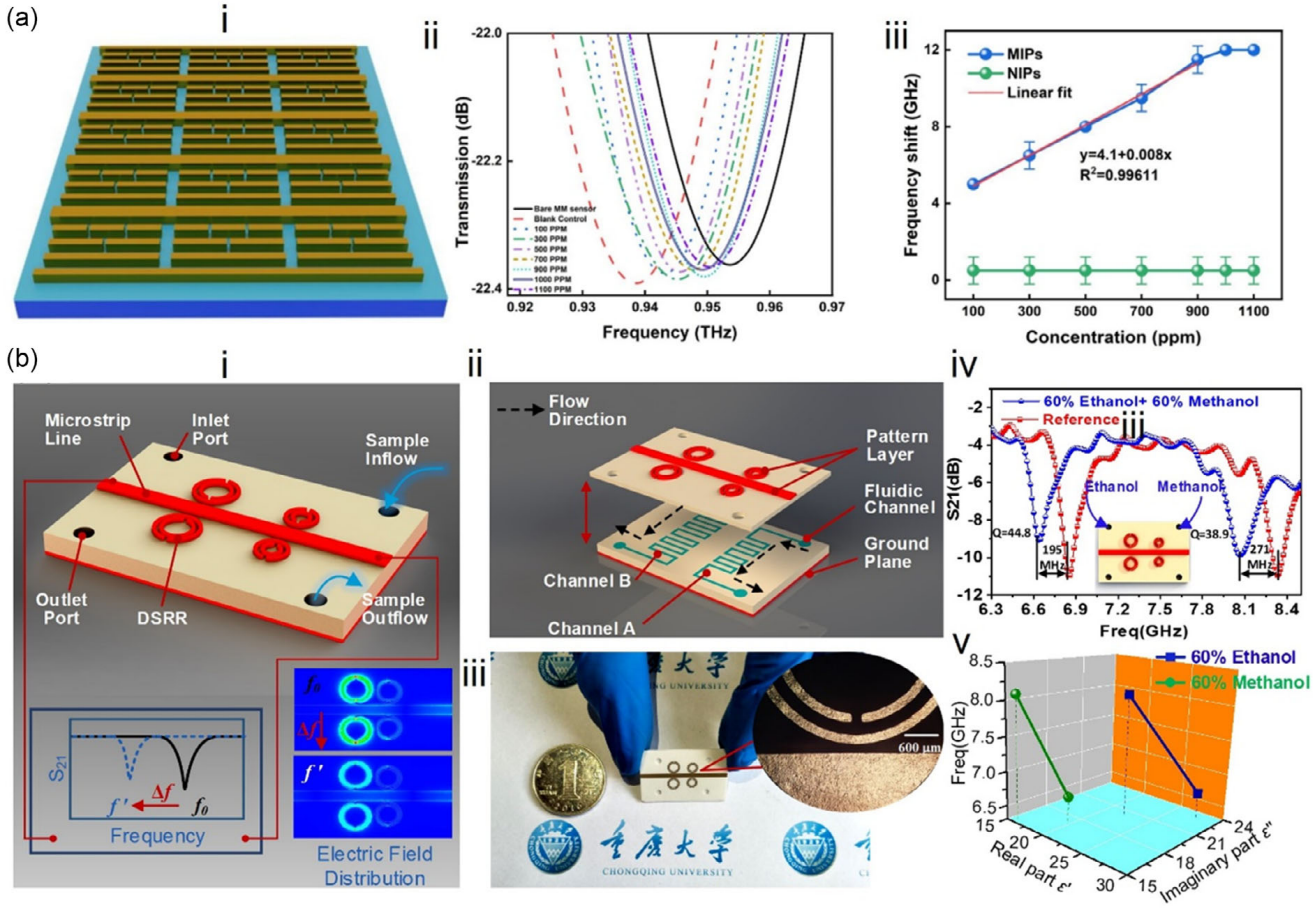
Resonant antennas for VOC detection in the terahertz and microwave region. a) Resonant terahertz sensor. i) Schematic diagram of molecule sensing. ii) Transmittance spectra of the THz sensor with different concentrations of analytes. iii) Plot showing the relationship of signal strength versus concentrations. Reproduced with permission.^[^ ^344^ ^]^ Copyright 2022, Elsevier. b) Resonant VOC sensor in the microwave region. i) Mock‐up view of the VOC sensor. ii) Exploded views. iii) Photograph of the sensor. iv) Spectral response of the sensor after the loading of VOCs. v) The dielectric characteristic line of VOCs obtained from the measured spectra. Reproduced with permission.^[^ ^132^ ^]^ Copyright 2018, Springer Nature.

THz antennas for gas sensing rely on the interaction of THz radiation with the resonant structures.^[^ ^336^, ^337^ ^]^ The resonant structures can be designed to have specific spectral responses that are sensitive to the presence of gas molecules, leading to a change in the THz signal, such as resonant frequency.^[^ ^338^, ^339^, ^340^ ^]^ The amount of signal change is related to the concentration of gas molecules in the sensing area, providing a means for VOC gas detection. THz antennas for gas sensing can be fabricated using various techniques, such as photolithography and nanoimprint lithography. The resonant structures in the antennas can be designed with different shapes and dimensions, such as split‐ring resonators, fishnet structures, and spiral structures.^[^ ^341^, ^342^, ^343^ ^]^ The antennas can also be coated with a selective material to enhance the selectivity of the sensor to specific VOC gases. THz antennas typically have subwavelength dimensions, ranging from tens to hundreds of microns. Guo and his team have devised a novel terahertz metamaterial sensor that employs molecularly imprinted polymers for highly selective detection of gaseous hexanal at ppm concentration levels (**Figure** **15**a(i)).^[^ ^344^ ^]^ When hexanal gas is introduced to the MIP‐modified sensor surface, it undergoes specific adsorption by the hexanal MIPs, which results in a noticeable blueshift in the resonance frequency of the sensor (Figure 15a(ii)). The modified sensor can sensitively detect hexanal gas in concentrations ranging from 100 to 900 ppm, with a remarkable maximum frequency shift of 12 GHz (Figure 15a(iii)).

In the microwave region, the dimensions of antennas are typically on the order of millimeters to centimeters^[^ ^345^, ^346^ ^]^ because the operating wavelength in the microwave region is on the order of centimeters to millimeters, which sets the scale for the size of the antennas. The integration of antennas and microfluidics at this scale exhibits notable advantages. The first is enhanced sensitivity.^[^ ^347^ ^]^ The integration of metamaterials with microfluidic channels can enhance the interaction between the electromagnetic waves and the analytes of interest, leading to increased sensitivity and detection limits. The second is device miniaturization.^[^ ^348^ ^]^ The use of microfluidic channels allows for the miniaturization of the sensing system, making it more portable and easy to integrate into other devices or systems. The third is real‐time detection.^[^ ^349^, ^350^ ^]^ The use of microfluidic channels allows for the continuous flow of analytes past the metamaterial sensing elements, enabling real‐time detection and monitoring of the analyte concentration. For instance, Zhou and co‐workers developed a microfluidics‐antenna‐integrated sensor for multiband sensing of VOCs’ dielectric properties (Figure 15b(i)).^[^ ^132^ ^]^ The sensor comprises several pairs of highly sensitive symmetrical double split‐ring resonators and microfluidic channels with a capacity of only 4 μL (Figure 15b(ii,iii)). They established and experimentally verified a dielectric model to accurately estimate the complex permittivity and achieve multiband sensing. Increasing the number of resonators in the sensor enables obtaining a dielectric spectrum for more detailed VOCs’ dielectric information (Figure 15b(iv,v)).

## 5 Intelligent and Integrated

### 5.1 Artificial Intelligence Enhances VOC Sensing

**Figure 16:**
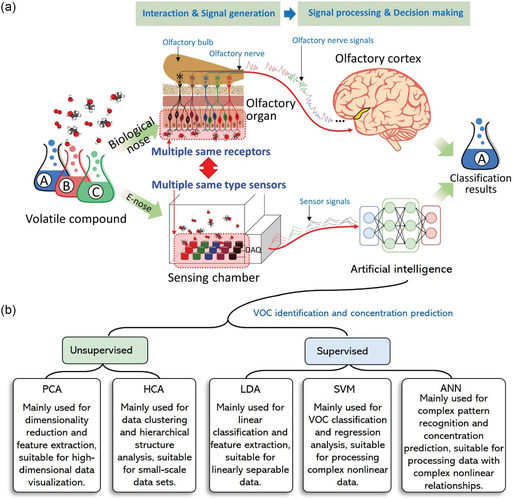
a) Comparison of the human olfactory system and the bionic nose. Reproduced with permission.^[^ ^354^ ^]^ Copyright 2023, PLOS. b) Artificial intelligence algorithms commonly used in artificial olfactory systems.

Humans identify odors through the olfactory system, which includes olfactory receptor cells within the nasal cavity connected to the nervous system. Olfactory receptor cells are located on the olfactory mucosa within the nasal cavity and contain odor receptors that interact with chemicals in the air. When these chemicals enter the nasal cavity, they react with the odor receptors, activating the olfactory receptor cells, which then transmit signals through neural pathways to the olfactory bulb in the brain. The olfactory bulb is a specialized region in the brain responsible for odor processing, receiving signals from olfactory receptor cells and relaying them to other parts of the brain, such as the cerebral cortex and limbic system. In the brain, these signals are interpreted and analyzed, allowing humans to perceive and identify different odors.^[^ ^351^, ^352^, ^353^, ^354^ ^]^ This mechanism also applies to artificial VOCs sensing and olfactory systems. In artificial olfaction systems, various types of sensors are used to detect volatile compounds in the environment. When volatile compounds are single components, direct measurement can be achieved through simple signal processing and data analysis. However, simple data processing may not suffice for sensing complex combinations of volatile compounds. Therefore, it is necessary to introduce artificial intelligence (AI) algorithms into the system to handle high‐dimensional pattern data and accomplish gas identification functions (**Figure** **16**a).^[^ ^107^, ^353^, ^355^, ^356^, ^357^, ^358^, ^359^ ^]^

In artificial olfaction systems, there are generally two categories of AI: unsupervised and supervised learning (Figure 16b).^[^ ^360^, ^361^ ^]^ The goal of unsupervised learning is to discover hidden patterns or structures from unlabeled data without any prior knowledge about data labels or outputs. The most widely used unsupervised learning method is principal component analysis (PCA). In VOCs sensing, PCA can be employed to process multidimensional VOCs data collected by sensors, aiding in the discovery of the primary features and structures within the data, thereby achieving dimensionality reduction and visualization of the data.^[^ ^362^, ^363^, ^364^ ^]^ The basic idea of PCA is to map the original data to a new, lower dimensional space through linear transformation, such that the mapped data exhibit the maximum variance. In this new lower dimensional space, each dimension represents a principal component of the original data, where the first principal component has the largest variance, the second principal component has the next largest variance, and so forth. By retaining principal components with larger variances, dimensionality reduction can be achieved while preserving as much information from the original data as possible. For example, Boutamine and colleagues demonstrated the application of PCA in VOC identification.^[^ ^364^ ^]^ In their work, a QCM coated with hexamethyldisiloxane was used to detect VOC molecules. They collected frequency changes of the QCM under different gas concentrations and used this data as a database, employing PCA for VOC identification. The results showed that in a low‐dimensional space, PCA could easily identify ethanol molecules. However, the effectiveness of PCA depends on the distribution and structure of the data. If different types of VOC data do not separate clearly in the principal component space, effective VOC identification becomes difficult. For instance, in Boutamine et al.'s work, it was challenging to distinguish chloroform and benzene using only PCA because their areas overlapped significantly in the principal component space. Additionally, PCA may lead to the loss of some important information during dimensionality reduction, affecting the accuracy of VOC identification. Therefore, it is often necessary to combine PCA with other methods to improve the accuracy of VOC identification.

Another popular unsupervised method for analyzing multivariate data is clustering analysis. Hierarchical clustering analysis (HCA) is a commonly used clustering technique that divides data samples into hierarchical categories, forming a dendrogram of clustered structures. In gas sensing, HCA can assist in identifying gas samples with similar features and organizing them into different levels of clusters to understand the relationships and structures among gas samples.^[^ ^365^, ^366^ ^]^ The advantages of HCA include not requiring a predetermined number of clusters and providing clustering hierarchy and visualization results. For example, Li et al. systematically analyzed the recognition of different VOCs by piezoelectric cantilever beam resonators using PCA and HCA.^[^ ^367^ ^]^ The results showed that the recognition accuracy of HCA for four VOCs was 87.5%. However, HCA has a high computational complexity, particularly when dealing with large‐scale data, which may pose challenges.

Compared to unsupervised learning, supervised learning requires labeled training data and is primarily used for prediction or classification tasks. The goal of supervised learning is to learn a function from labeled training data that can map input data to predefined output labels or categories. In supervised learning, each training sample consists of an input feature vector and its corresponding output label or category. The learning algorithm analyzes these data to build a model, enabling predictions or classifications for new, unseen data. Common supervised learning methods in gas component analysis include linear discriminant analysis (LDA), support vector machines (SVM), and artificial neural networks (ANN).

LDA is a linear statistical technique. LDA shares some similarities with PCA in that both construct a set of orthogonal dimensions composed of linear combinations of the original dimensional space, and both methods reduce dimensionality by preserving multiple dimensions that meet threshold criteria. However, LDA is a supervised learning method that requires labeled training data to distinguish differences between different categories. Additionally, the objective of LDA is to maximize the differences between categories while reducing the dimensionality of the data, i.e., to maximize the distance between different categories after projection into a lower dimensional space while minimizing the variance within categories. This contrasts with PCA, which aims to retain the maximum variance in the data without considering class labels. In recent reports, Qu et al. developed a nondestructive, high‐throughput gas sensor based on SERS to identify and quantify multiple VOCs in muscle foods.^[^ ^368^ ^]^ To demonstrate the gas sensor's capability in assessing food freshness, Qu et al. used LDA to process the spectral information. Based on the VOC fingerprints obtained by the gas sensor, the LDA model achieved an accuracy of 96.9% in classifying fresh, less fresh, and spoiled samples. This high accuracy showcases the potential of LDA in practical applications, particularly in handling and distinguishing complex classification tasks.

SVM is a widely used nonlinear statistical technique applied to classification and regression tasks. Its core idea is to find a hyperplane that maximizes the margin between different classes of data points, effectively separating them. When dealing with nonlinear problems, SVM can map data to a high‐dimensional feature space using kernel tricks or kernel properties, thereby finding a linearly separable hyperplane in the high‐dimensional space. Commonly used kernel functions include linear kernel, polynomial kernel, Gaussian kernel, and so on, which can map data from the original feature space to a higher dimensional space for classifying nonlinear problems. In the field of gas sensing, SVM can be utilized for tasks such as gas sample classification, anomaly detection, gas concentration prediction, and so on, aiding in feature extraction from gas sensor data and facilitating effective analysis and application of gas data.^[^ ^369^ ^]^ For instance, Wang et al. used a QCM gas sensor array and SVM to detect infestations of tree trunks by borers.^[^ ^370^ ^]^ Four plant VOCs (α‐pinene, β‐phellandrene, 3‐carene, and cis‐thujopsene) were used as biomarkers of whether tree trunks were infested by borers. In their sensor demonstration, the frequency shifts of the QCM were used as feature values. These feature values were used to construct a feature matrix. The response matrix was visualized using PCA and LDA before further classification with SVM. The results showed that SVM exhibited satisfactory recognition accuracy in both the calibration set (97.62%) and the validation set (93.75%). This demonstrates the versatility and effectiveness of SVM in VOC sensing applications. By leveraging advanced techniques such as the kernel trick, SVM can handle complex, nonlinear classification tasks, making it a powerful tool for analyzing and interpreting gas sensor data.

ANN is a computational model that mimics the structure and functionality of biological neural networks, consisting of many neurons (or nodes) connected together. Each neuron receives multiple inputs and produces an output. ANN typically consists of input layer, hidden layers, and output layer, with the hidden layer potentially containing one or more levels. ANN methods are powerful and flexible for modeling nonlinear problems. However, this flexibility is also a major weakness of artificial neural networks. Careless design (i.e., “overfitting”) or insufficient training of the network can easily lead to learning and predicting unnecessary or nonexistent nonlinear behaviors. ANN are also a biomimetic technology used to classify and predict results based on the similarity and proximity between complex and nonlinear data. Currently, probabilistic neural network, radial basis function network, backpropagation neural network (BPNN), and convolutional neural networks (CNN) have been used for VOC classification.^[^ ^109^, ^371^, ^372^ ^]^ As a typical example, Thrift et al. proposed an odor compass based on SERS sensors and machine learning to locate multiple chemical sources and pathogens.^[^ ^373^ ^]^ Specifically, Thrift et al. implemented a sensor grid array to identify small spatial variations in analyte chemical adsorption and used various machine learning models to identify multiple analyte source directions. Experimental results showed that CNN and SVM classifier models achieved over 90% accuracy for the multiodor source problem. Subsequently, the system was applied to pinpoint the location of *Escherichia coli* biofilms through the identification of VOCs.

### 5.2 Electronic Nose

**Figure 17:**
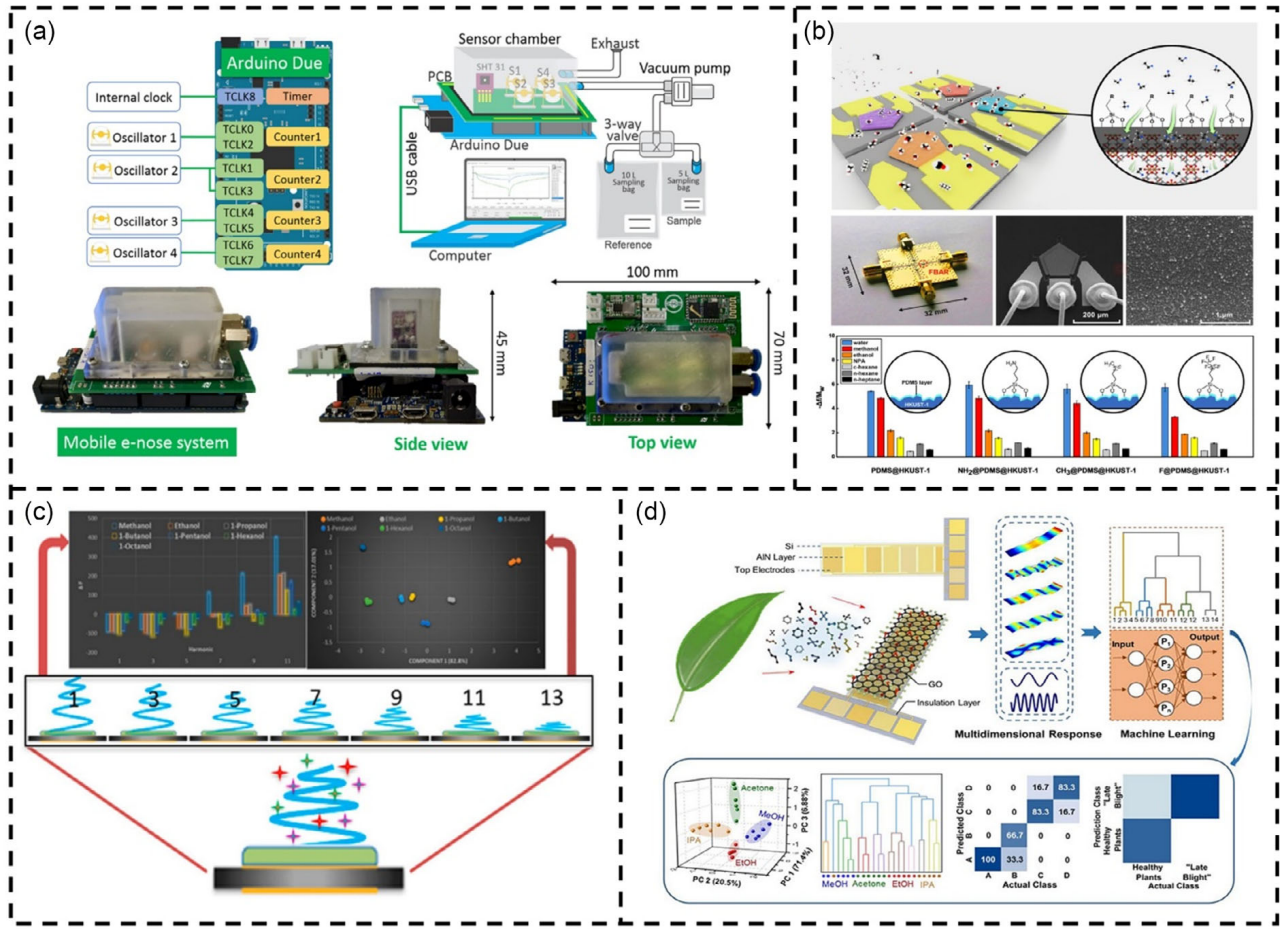
E‐nose technology based on nanomechanical sensors. a) A mobile QCM‐based e‐nose equipped with a data acquisition (DAQ) system. Reproduced with permission.^[^ ^374^ ^]^ Copyright 2020, American Chemical Society. b) Schematic of the FBAR sensor array (four devices) for vapor detection. Reproduced with permission.^[^ ^385^ ^]^ Copyright 2020, American Chemical Society. c) QCM‐based VSA for VOC vapor identification. Reproduced with permission.^[^ ^386^ ^]^ Copyright 2015, American Chemical Society. d) Schematic illustrations of the proposed VSA based on piezoelectric cantilever for plant diseases diagnosis. Reproduced with permission.^[^ ^367^ ^]^ Copyright 2022, American Chemical Society.

Due to the diversity of VOCs, a single MEMS resonator cannot meet the application requirements. To address this, researchers have expanded the device from a single sensor to a multisensor array (MSA), aiming to detect various VOCs with a single system. The strategy of using a series of devices with several different sensitizers to detect multiple analytes is common in gravimetric vapor sensing research. The resulting device is often referred to as an electronic nose or “e‐nose” because it attempts to mimic the function of the human nose.^[^ ^43^ ^]^ For example, Julian et al. designed a low‐cost mobile e‐nose system using a QCM sensor array.^[^ ^374^ ^]^ This sensing system consists of a sensor array of four QCMs. Different polymer films (i.e., polyacrylonitrile (PAN), PVDF, poly(vinyl pyrrolidone) (PVP), and poly(vinyl acetate) (PVAc)) were spin‐coated on the surface of each device to accurately identify multiple VOCs (**Figure** **17**a). To collect the sensing dataset, Julian et al. developed a multichannel data acquisition circuit and calibrated it using a function generator. Subsequently, the sensing performance of the four prepared QCM sensors was validated by exposing them to seven different VOCs. The results showed that the differently functionalized QCMs exhibited varying levels of sensitivity to different VOC gases, with high reproducibility and consistency. To further enhance sensitivity and identification accuracy, different sensitive films, larger QCM arrays, and more advanced algorithms have been successively introduced.^[^ ^167^, ^375^, ^376^, ^377^ ^]^

Currently, MSA is also being applied to other MEMS‐based gravimetric sensing technologies.^[^ ^378^, ^379^, ^380^, ^381^, ^382^ ^]^ For example, Ahmed et al. described a sensor device comprising an array of eight polymer‐coated piezoelectric microcantilevers (two each coated with polyacrylic acid, PEI, and polyethylene glycol, along with two uncoated cantilevers) and an electronic resonant frequency readout.^[^ ^383^ ^]^ By measuring the frequency shift of the resonant microcantilevers, they demonstrated the sensor system's ability to distinguish between acetone, ethanol, and octane, with detection limits of 1568, 383, and 87 ppmv, respectively. Benetti et al. proposed an e‐nose based on SAW for detecting VOCs (such as ethyl acetate) and chemical warfare agent simulants (such as DMMP, dichloromethane (DCM), and dichloropentane (DCP)).^[^ ^184^ ^]^ This system consists of six SAW resonators coated with five different polymers and one uncoated SAW device used as a reference. The SAW e‐nose was tested by exposing it to ethyl acetate, DMMP, DCM, DCP, and water vapor. The results show that the SAW e‐nose allows discrimination of all considered vapors within the tested concentration range. Mahmud et al. proposed a low‐power wearable e‐nose system based on a CMUT array.^[^ ^384^ ^]^ This e‐nose system comprises a 5‐channel CMUT sensor array, with each CMUT sensor coated with a different receptor material to detect four volatile compounds: ethanol, toluene, p‐xylene, and styrene. The resolution of all channels in the sensor array was below 10 ppm. Finally, appropriate pattern recognition techniques were employed to identify VOC gases.

Although numerous MEMS sensor arrays have been used for VOC identification, their lower operating frequencies limit their sensitivity. FBAR sensors, with typical operating frequencies in the gigahertz range, offer higher sensitivity according to the Sauerbrey equation. Additionally, FBAR devices have smaller sizes, making them more suitable for system integration and miniaturization. For instance, Lu et al. demonstrated that an FBAR sensor array functionalized with four types of supramolecular monolayers could selectively detect various VOCs.^[^ ^74^ ^]^ Following this, Yan et al. utilized an FBAR sensor array integrated with four different MOF materials to detect methanol, ethanol, n‐propanol (NPA), n‐hexane, cyclohexane (c‐hexane), and n‐heptane vapors (Figure 17b).^[^ ^385^ ^]^ The results showed that the FBAR sensor array can detect a variety of trace vapors with high sensitivity (detection limit as low as 1 ppm).

Although MSA can identify and differentiate unknown gases through multidimensional features and pattern recognition techniques, these systems also have drawbacks such as large size, complex sensing circuits, and high failure rates.^[^ ^386^ ^]^ To overcome these disadvantages, a new mechanism called the virtual sensor array (VSA) has been developed.^[^ ^363^, ^387^, ^388^, ^389^, ^390^ ^]^ This mechanism uses a single sensor to generate a multidimensional output vector similar to that of an e‐nose. The output vector is then processed using pattern recognition algorithms to accurately identify different VOCs. Compared to MSA, VSA offer significant advantages, including lower cost, simpler systems, and smaller size. The key to forming a VSA is to achieve different response patterns within a single device. A common approach is to exploit the intrinsic response patterns of specific sensors. For example, Speller et al. used multiple harmonics of a QCM as VSA. The QCM surface was coated with a thin layer of ionic liquid as a sensitizer to adsorb different VOC gases. As a proof of concept, the QCM VSA was exposed to 18 organic vapors, including alcohols, hydrocarbons, chlorinated hydrocarbons, and nitriles, which belonged to the same or different homologous series. The results showed that the QCM VSA could classify all 18 test analytes with nearly 100% accuracy. Furthermore, higher order resonance modes can also be used to construct a VSA.^[^ ^391^, ^392^ ^]^ However, some higher order resonance modes exhibit lower resonance peak amplitudes, leading to lower signal‐to‐noise ratios and sensor sensitivity, which, in turn, result in poorer detection limits.

Another method to construct a VSA is through device design or external modulation. In terms of structural design, Li et al. designed a piezoelectric cantilever with five sets of top electrodes on the cantilever.^[^ ^367^ ^]^ Different sets of top electrodes were used to enhance the amplitude of resonance peaks for multiple modes of the cantilever. Subsequently, a GO film was deposited on the cantilever surface to form a VOC sensor. The frequency shifts and impedance changes of multiple resonance modes were considered the VSA's response to VOCs, generating unique fingerprints for each VOC. Using machine learning algorithms, the proposed VSA could accurately identify different types of VOCs and mixtures, achieving accuracies of 95.8% and 87.5%, respectively. For external modulation, temperature modulation is the most widely adopted method. For example, Zeng et al. designed and fabricated a single‐chip temperature‐compensated FBAR for detecting and identifying various VOCs.^[^ ^389^ ^]^ In their work, a programmable heater was added beneath the FBAR chip to achieve multiparameter VSA. By measuring the frequency and impedance responses at different temperatures, they obtained multidimensional information about the analytes. Finally, PCA and LDA were used for evaluation, and the results showed that all analytes could be distinguished and classified with over 97% accuracy. Although VSA can collect multidimensional information using a single device, there are still issues with cross‐reactivity between multiple VOCs and the sensitive films. In the future, combining VSA and MSA methods is expected to balance sensor size and cross‐reactivity, thereby achieving higher detection precision and identification accuracy for VOCs.

### 5.3 Photonic Nose

Combining nanophotonics with AI enables the development of a photonic nose. As described in the previous section, the main sensing mechanisms for optical gas sensors can be categorized into those based on RI and those based on absorption. Here, RI sensors primarily detect the real part of the RI. VOC sensors based on RI rely on the RI changes induced by analytes, leading to resonance wavelength shifts, commonly observed in near‐IR (NIR), terahertz, and microwave spectra. However, distinguishing between different analytes solely based on RI changes is challenging. Therefore, this approach lacks selectivity. To address this issue, surface functionalization is often employed to enhance the specificity of photonic devices, albeit at the cost of added manufacturing complexity. Another approach to improve the poor selectivity of RI sensors is to detect the imaginary part of the RI. The imaginary part of the RI represents the medium's absorption capacity. In photonic devices based on metasurfaces, changes in the imaginary part of the RI are typically reflected as variations in absorption amplitude. Introducing changes in absorption amplitude as additional information into photonic sensors enables the construction of complex RI sensing devices. This additional information also enhances the selectivity of photonic devices. For example, Fu et al. proposed a terahertz metasurfaces based on the electromagnetically induced transparency (EIT) effect for sensing and classifying three different VOCs (ethylbenzene, isopropanol, and ethyl acetate).^[^ ^393^ ^]^ For pure VOCs in the liquid phase, changes in both the resonance frequency and amplitude information of the THz metasurface occurred as their volume increased. Leveraging both resonance frequency and amplitude information, combined with machine learning algorithms, successfully differentiated between different VOCs.

**Figure 18:**
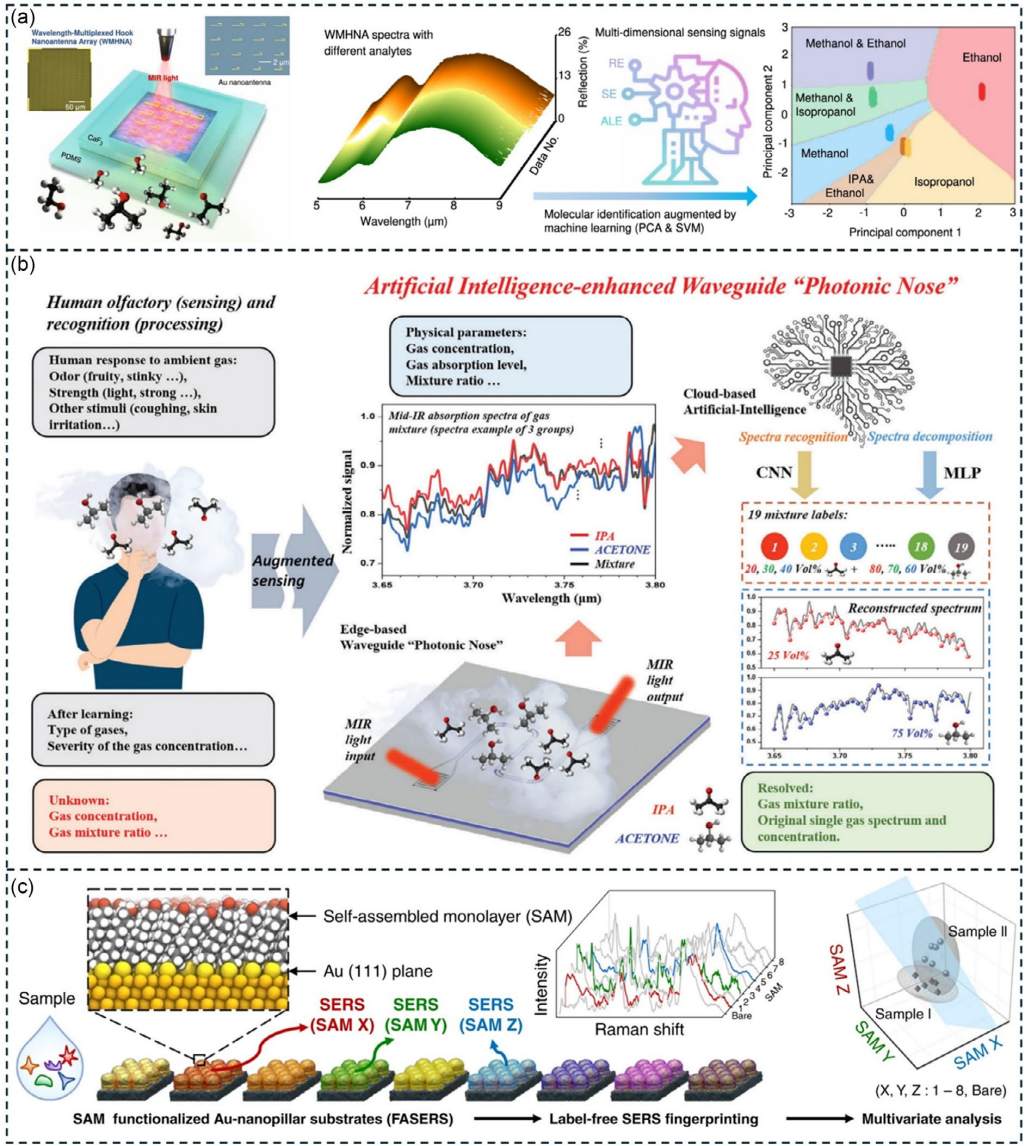
Nanophotonic nose. a) WMHAN for sensing various VOCs. Machine learning process to extract multidimensional sensing information for recognition of 1% alcohols and their mixtures in water. Reproduced with permission.^[^ ^362^ ^]^ Copyright 2022, Springer Nature. b) AI‐enhanced waveguide “photonic nose”‐augmented sensing platform for VOC gases in mid‐IR. Reproduced with permission.^[^ ^395^ ^]^ Copyright 2024, John Wiley and Sons. c) SERS‐based artificial nose for high‐dimensional fingerprint recognition. Reproduced with permission.^[^ ^397^ ^]^ Copyright 2020, Springer Nature.

In the mid‐IR band, optical absorption related to the imaginary part of the RI may be more intriguing. This band encompasses characteristic absorption peaks of various VOCs. These absorption peaks can offer additional dimensions of information for molecular identification and quantitative analysis. The introduction of metasurfaces and machine learning further enhances the detection limits of traditional IR spectroscopy techniques. Ren et al. proposed a wavelength‐multiplexed hooked nanoantenna array (WMHNA) for molecular identification of liquid‐phase mixed alcohols (methanol, ethanol, and isopropanol) (**Figure** **18**a).^[^ ^362^ ^]^ In this work, a nanoantenna array with ultrawideband operating wavelengths (6–9 μm) was constructed within an extremely large unit cell by varying the dimensions of the hooked nanoantennas gradiently. The ultrawideband operating wavelengths enable the simultaneous collection of more fingerprint vibrational information. Based on the multidimensional spectral information collected on the WMHNA platform, the authors effectively classified the multidimensional information using machine learning algorithms (PCA and SVM). The results indicate a 100% accuracy in alcohol molecule identification using WMHNA. Currently, machine learning‐enhanced sensing schemes based on metamaterials have been widely employed in fields such as molecular identification and dynamic reaction monitoring.

Subsequently, Zhou et al. introduced an AI‐enhanced metamaterial waveguide sensing platform for the analysis of aqueous mixtures in the mid‐IR range.^[^ ^394^ ^]^ SWG metamaterials were utilized to enhance the sensitivity of the waveguide sensor within a small footprint area. Leveraging machine learning, this platform successfully distinguished the mid‐IR absorption spectra of ternary mixtures in water (acetone, IPA, and glycerol) and decomposed them into individual component spectra for concentration prediction. Specifically, Zhou et al. initially employed a CNN to classify spectra of mixtures with 64 predefined ratios, achieving a classification accuracy of 98.88%. Next, an multilayer perceptron (MLP) regressor was applied to the 64 mixture spectra for spectral decomposition and concentration prediction. The results indicated that 81% of the predicted values were within a root mean square error (RMSE) range of 0.1 vol%.

As a significant demonstration for mid‐IR VOC gas mixtures, Liu et al. proposed an AI‐assisted waveguide “photonic nose” (Figure 18b).^[^ ^395^ ^]^ This platform utilized a subwavelength grating cladding design to create a suspended structure and optimized sensitivity for enhanced sensing applications. The authors employed two machine learning algorithms to analyze the absorption spectra of gas mixtures (acetone and IPA). First, CNN was utilized to classify gas mixtures with 19 different binary mixture ratios, achieving a classification accuracy of 93.57%. Subsequently, an MLP regressor was used for spectral decomposition of the original pure spectra of gas mixtures. The spectral decomposition and concentration prediction of gas mixtures showed an average RMSE of 2.44 vol%. This work demonstrates that “photonic nose” can provide quantitative sensing and analysis capabilities, paving the way for more complex multigas mixture analysis in on‐chip spectral sensing systems.

Similar to IR spectroscopy, introducing machine learning into SERS can also achieve rapid classification and identification of analytes. Currently, this approach is widely used for VOCs identification.^[^ ^290^, ^368^, ^373^ ^]^ Qiao et al. demonstrated a SERS platform for monitoring gaseous aldehydes in breath components.^[^ ^396^ ^]^ However, SERS sensitivity is severely limited due to inadequate contact between high‐speed flowing gas molecules and the solid substrate. To enhance the adsorption of gas molecules, Qiao et al. coated a layer of ZIF‐8 on gold superparticle (GSP) self‐assemblies to slow down the flow rate of gas biomarkers. This method effectively slows down the gas flow rate to allow sufficient time for analytes to adsorb onto the SERS substrate. Subsequently, gaseous aldehydes (benzaldehyde, salicylaldehyde, and glutaraldehyde) were selectively captured onto the GSP substrate through Schiff base reactions. Then, PCA was employed to classify the collected signals, resulting in excellent classification performance.

While SERS offers hope for VOCs identification with the additional spectral dimension it provides, highly overlapping spectra may hinder sample identification in complex biological systems. To address this issue, it is necessary to construct SERS sensor arrays and functionalize specific receptors on each array to enhance selectivity. This approach enables SERS to embrace more spectral data (also known as SERS superprofile), thereby significantly improving the accuracy of biological sample identification. For instance, Kim and colleagues developed a SERS artificial nose for high‐dimensional fingerprint recognition (Figure 18c).^[^ ^397^ ^]^ The array‐based artificial nose sensing platform comprises eight different SAM‐functionalized gold nanorod substrates and one bare unmodified substrate. These SAMs exhibit a range of nontargeted and mild selectivity to facilitate various physical and chemical interactions with different samples. The mild selectivity of SAMs can influence the strength and configuration of analyte interactions with the plasma surface, thereby diversifying the resulting SERS fingerprints. Compared to traditional label‐free SERS, the reliance on SAM‐based multidimensional spectral datasets enables the identification and differentiation of complex biological samples with higher accuracy. Finally, discrimination of multidimensional spectral data using PCA resulted in 100% identification accuracy. This work provides new insights into high‐dimensional fingerprint recognition based on SERS.

### 5.4 Miniaturized Photonic Platform

The complexity of optical testing systems is a key limitation that hinders the rapid development of photonics noses. To overcome this limitation, building miniaturized optical measurement systems has become increasingly critical.^[^ ^398^ ^]^ Miniaturized optical platforms are expected to spark more applications in fields such as sensor networks and the IoT. However, directly scaling down optical systems poses significant challenges. As optical systems become more compact, the path length necessarily decreases, leading to reduced spectral resolution. In recent years, on‐chip waveguide technology has been proposed as one of the paths to miniaturizing spectroscopic systems.^[^ ^257^, ^399^, ^400^, ^401^ ^]^ This technology allows for more compact optical confinement to further reduce footprint without severely compromising performance. For example, Qiao et al. demonstrated an on‐chip computational spectrometer in mid‐IR (3.7–4.05 μm) using an MEMS‐enabled silicon photonic integrated device.^[^ ^126^ ^]^ This experimental setup acquires spectra through the temporal driving of MEMS‐tunable waveguide couplers. Subsequently, machine learning regularized regression methods are employed to compute the reconstructed spectra. The spectral resolution of the showcased Si‐McS is determined to be 3 nm based on dual‐wavelength reconstruction results. Finally, a chamber filled with nitrous oxide (N_2_O) gas is introduced into the experimental setup to examine the absorption spectral reconstruction capability of Si‐McS. Experimental results demonstrated that the spectral absorption characteristics of N_2_O can be effectively reconstructed and identified. This work presents a novel solution for achieving miniaturized, robust, and cost‐effective on‐chip spectroscopic sensing systems.

**Figure 19:**
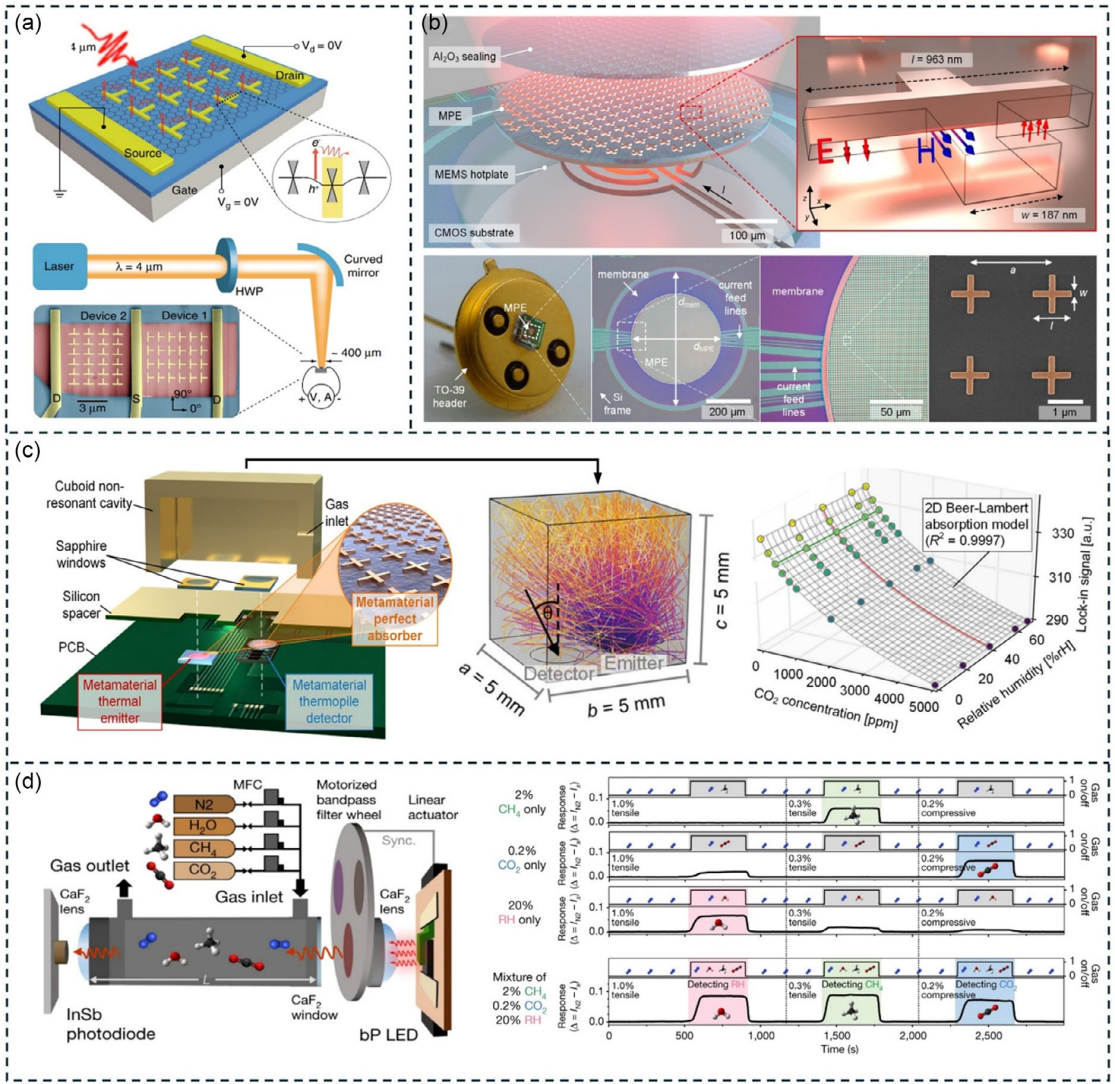
Miniaturized spectrometer for VOC sensing. a) Zero‐bias mid‐IR graphene photodetectors. Reproduced with permission.^[^ ^406^ ^]^ Copyright 2020, Springer Nature. b) On‐chip metamaterial thermal emitter. Reproduced with permission.^[^ ^412^ ^]^ Copyright 2017, American Chemical Society. c) All‐metamaterial gas sensor. Reproduced with permission.^[^ ^413^ ^]^ Copyright 2020, American Chemical Society. d) Nondispersive IR gas sensing system using a strain‐tunable BP light‐emitting diodes. Reproduced with permission.^[^ ^414^ ^]^ Copyright 2021, Springer Nature.

The unique wavefront manipulation capabilities of metasurfaces also present significant opportunities for miniaturizing optical measurement systems.^[^ ^402^, ^403^, ^404^, ^405^ ^]^ Specifically, selectively transmitting light of specific wavelengths based on metasurfaces enables the construction of narrowband filters. Compared to spectrographs based on dispersion systems, spectrometers based on narrowband filters have key advantages in miniaturization. In addition to the advantages of planarity, there is no need for separation (i.e., path length) between the spectral filtering elements and detectors, thus circumventing one of the fundamental limitations of dispersive devices and providing more possibilities for compact systems. For the integration of photodetectors, Wei et al. proposed metasurface‐mediated graphene photodetectors (MMGPD) for polarization detection (**Figure** **19**a).^[^ ^406^ ^]^ These photodetectors exhibit polarization‐correlated photocurrents generated by artificially induced anisotropy from carefully designed asymmetrically broken nanoantennas. Notably, MMGPDs demonstrate high responsivity and low noise equivalent power, reaching as low as 0.12 nW Hz^−1/2^ at zero bias. Currently, metasurface‐based methods have been widely used in photoelectric detection, including polarized and spin light.^[^ ^255^, ^407^ ^]^ Based on these works, Xie et al. further extended the operating wavelength of MMGPD to the long‐wave IR for integration into a spectroscopic sensing platform.^[^ ^408^ ^]^ Long‐wave IR holds immense potential in chemical/biological sensing as it covers rich absorption fingerprints of gas molecules, serving as biomarkers for healthcare monitoring and early disease diagnosis. To demonstrate the device's progress in molecular detection, Xie et al. selected acetone as the analyte for spectroscopic sensing. Experimental results revealed that the device has a lower detection limit of acetone vapor of 115 ppm and exhibits a fast dynamic response of 6 s. These results reveal the potential of the device as a multifunctional on‐chip miniature optoelectronic platform for polarization and spectral sensing, enabling real‐time environmental monitoring and biomedical screening.

Integrating metasurfaces to construct narrow‐band light sources is also a key component of low‐cost, compact mid‐IR gas sensing systems.^[^ ^409^, ^410^, ^411^ ^]^ Lochbaum et al. proposed an on‐chip narrowband thermal emitter suitable for the mid‐IR wavelength range by combining a MEMS heater with a metasurface (Figure 19b).^[^ ^412^ ^]^ This emitter exhibited a resonant emissivity of 0.99 and a quality factor of 15.7 at a central wavelength of 3.96 μm, with temperature stability (resonant wavelength shift of 0.04 nm °C^−1^) and emission characteristics independent of angle (up to 50°). Such an emitter holds promise for highly integrated free‐space optical gas sensing applications. Subsequently, the authors demonstrated the potential of this emitter in CO_2_ detection. Following this, the team presented the concept of an all‐metasurface gas sensor (Figure 19c).^[^ ^413^ ^]^ In this concept, metasurfaces act as on‐chip filters integrated separately into the thermal emitter and detector. Leveraging the filtering function of metasurfaces, this gas sensor selectively responds to specific target gases (in this case, CO_2_). Compared to traditional gas sensors, the prototype sensor reduces the absorption volume by 30 times. Notably, the filtering band of the metasurface can be tuned by modifying the structure of the metasurface. This work paves the way for various gas detection and even spectroscopic applications on a single substrate.

In addition to metasurfaces, the tunability of low‐dimensional materials also presents opportunities for miniaturization and spectral reconstruction of spectrometers. Kim et al. utilized the strain‐tunable bandgap of black phosphorus (BP) to develop actively tunable IR optoelectronic devices at room temperature, including LEDs and photodetectors (Figure 19d).^[^ ^414^ ^]^ Due to the highly strain‐sensitive nature of the BP bandgap, the detection range of photodetectors can be expanded by applying strain. Furthermore, the author demonstrated such applications of this tunable optoelectronic platform by performing multiple gas sensing experiments (CO_2_, methane, and water vapor). In the same year, Yuan et al. reported a novel approach to mid‐IR spectroscopy using a single tunable BP photodetector, with an effective area of only 9 × 16 μm^2^.^[^ ^415^ ^]^ The core component of this spectrometer is the BP detector based on a dual‐gate transistor configuration. The optical response of the photodetector can be significantly adjusted by changing the applied electric field. With the performance obtained from the BP photodetector, spectral measurements were carried out via computational methods. The entire spectral measurement process does not require optical components such as gratings, additional spectrometers, or tunable laser sources. Finally, the authors demonstrated the potential of this spectrometer in CO_2_ detection. Looking ahead, further advancements in tunable low‐dimensional materials could revolutionize the field of spectroscopy, enabling more compact and versatile spectrometers for various applications.

## 6 Application

This section focuses on the application areas of electronic noses and photonic noses. To better categorize the discussion, we have divided the applications into three parts based on whether they are related to living organisms: human health monitoring, plant health monitoring, and IoT (nonliving entities) monitoring. The following is a detailed introduction to each application area.

### 6.1 Medical Diagnosis and Health Monitoring

VOCs are crucial biomarkers that reflect an individual's physiological health status. Extensive research has revealed potential associations between VOC levels and chronic diseases,^[^ ^416^, ^417^ ^]^ cancers,^[^ ^418^, ^419^ ^]^ and infectious diseases.^[^ ^420^, ^421^ ^]^ Therefore, monitoring VOCs produced by the human body can facilitate early disease diagnosis, respiratory disease management, metabolic disease management, and infection monitoring. According to a thematic overview, the human body contains 1849 types of VOCs,^[^ ^422^ ^]^ distributed in breath, skin, urine, blood, saliva, sweat, tears, breast milk, and feces. Among them, there are 872 and 532 types of VOCs in breath and skin, respectively.^[^ ^423^ ^]^ These VOCs in the human body are divided into endogenous and exogenous types. Endogenous VOCs originate from normal and abnormal metabolic processes occurring within the body, which can be released into the environment through the lungs.^[^ ^424^ ^]^ Exogenous VOCs come from outside the body and enter the body through inhalation, ingestion, skin absorption, or other means.^[^ ^425^ ^]^

**Figure 20:**
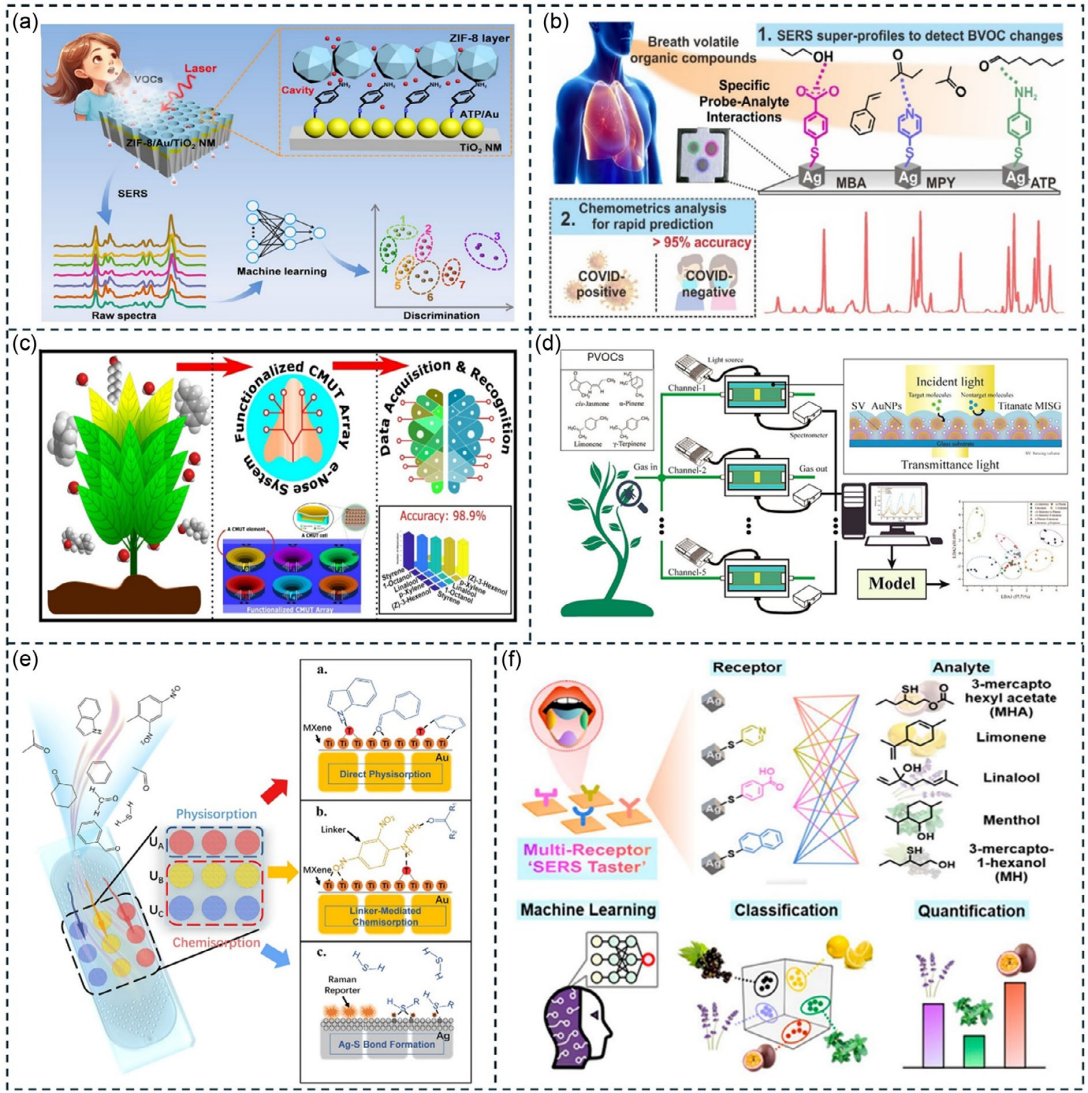
The applications of VOC sensors range from medical diagnosis, plant monitoring to the IoT. a) SERS substrate selective detection of aldehyde biomarkers in exhaled breath of lung cancer patients. Reproduced with permission.^[^ ^432^ ^]^ Copyright 2023, American Chemical Society. b) Noninvasive breath analyzer based on SERS. The analyzer uses VOCs in breath to identify COVID‐positive individuals. Reproduced with permission.^[^ ^289^ ^]^ Copyright 2022, American Chemical Society. c) Electronic nose system based on a functionalized CMUT array for selective detection of plant volatiles. Reproduced with permission.^[^ ^108^ ^]^ Copyright 2021, Elsevier. d) LSPR‐based sensor array for identification of plant biomarkers. Reproduced with permission.^[^ ^448^ ^]^ Copyright 2018, American Chemical Society. e) SERS‐based gas sensor for indoor air pollution monitoring. Reproduced with permission.^[^ ^452^ ^]^ Copyright 2022, American Chemical Society. f) SERS‐based taster for wine flavor molecular identification and quantification. Reproduced with permission.^[^ ^457^ ^]^ Copyright 2021, American Chemical Society.

Breath analysis is the primary method for monitoring VOCs. Major VOCs in the breath of healthy individuals include acetone (1.2–900 ppb), ethanol (13–1000 ppb), methanol (160–2000 ppb), isoprene (12–580 ppb), ammonia, and others.^[^ ^426^ ^]^ Differences between individuals can affect the concentration ratios of VOCs in their breath compositions. For instance, smokers tend to have higher concentrations of acetonitrile in their exhaled breath.^[^ ^427^ ^]^ Elevated isoprene concentrations in exhaled breath are often associated with exertion. During exertion, the concentration of isoprene in exhaled breath can increase 2–5 times.^[^ ^428^ ^]^ Elevated levels of ethane and pentane are associated with chronic lung diseases.^[^ ^429^ ^]^ Specific VOC levels can also be utilized for cancer diagnosis. For example, Asimakopoulos et al. used a gravimetric sensor coated with metalloporphyrins to identify prostate cancer by detecting VOCs in the headspace of urine.^[^ ^430^ ^]^ Bartolazzi et al. used a similar approach to analyze VOCs in the urine headspace to diagnose urinary tract cancers.^[^ ^431^ ^]^ Xu et al. developed a highly sensitive SERS substrate for selectively detecting aldehyde biomarkers in the exhaled breath of lung cancer patients (**Figure** **20**a).^[^ ^432^ ^]^ This SERS substrate consisted of Au/TiO_2_ and surface‐modified ZIF‐8, with ZIF‐8 acting as a gas trapping cavity for gas enrichment. Benzaldehyde was used as a typical gas marker model for lung cancer. Experimental results demonstrated ppb‐level detection sensitivity of benzaldehyde using this SERS substrate. Machine learning combined with Raman spectroscopy was employed for distinguishing between types of gaseous aldehydes.

Breath composition monitoring also provides a crucial tool for diagnosing infectious diseases and controlling pandemics. In 2019, the COVID‐19 pandemic posed significant threats to the global economy and human health. One effective strategy for controlling the COVID‐19 pandemic is the development of highly accurate methods to rapidly identify and isolate SARS‐CoV‐2 infected patients. Respiratory droplet transmission is the primary mode of transmission for COVID‐19. Therefore, timely and accurate detection methods for viruses in breath and saliva are essential. To address this, Li et al. proposed a scheme that combines SEIRA with microfluidics for measuring COVID‐19 in breath and saliva.^[^ ^433^ ^]^ Additionally, Zhou et al. developed AI‐enhanced plasmon–phonon nanoantennas for the image‐guided screening of SARS‐CoV‐2 in infected individuals, spanning from symptom onset to virus clearance.^[^ ^434^ ^]^ This advancement provides invaluable insights into virus–host dynamics, facilitating the development of targeted treatments and preventive measures. Recent studies have indicated that the levels of VOCs such as aldehydes, ketones, and alcohols in breath can serve as specific biomarkers for COVID‐19.^[^ ^435^, ^436^ ^]^ Leong et al. designed a SERS‐based breath analyzer to distinguish positive individuals from healthy people (Figure 20b).^[^ ^289^ ^]^ By functionalizing various molecular receptors on SERS, the sensor's discriminative ability is enhanced. Different molecular receptors interact with various VOCs in exhaled breath to generate information‐rich superprofile. Subsequently, a partial least squares discriminant analysis model is used for rapid, high‐throughput classification of COVID‐19 infection status. Finally, in a cohort of 501 participants, the breath analyzer achieved 95% sensitivity and specificity.

### 6.2 Plant Volatiles Detection

Real‐time, in situ monitoring of plant physiology is crucial for sustainable agriculture. Typically, the condition of plants can be understood by monitoring their growth status, surface temperature and humidity, hormones, and volatile substances.^[^ ^437^, ^438^, ^439^, ^440^, ^441^ ^]^ Among these, volatile substances are considered important information for understanding plant physiological status. Currently, more than 1,700 volatile compounds have been discovered from more than 90 plant families.^[^ ^442^ ^]^ These volatiles are mainly represented by terpenoids, phenylpropanoids/phenolics, fatty acid derivatives, and amino acid derivatives.^[^ ^443^ ^]^ In nature, the release of VOCs by plants is often regarded as a defense mechanism. It can be interpreted as induced resistance, where plants release volatile compounds after mechanical damage or insect feeding.^[^ ^444^ ^]^ For example, tomato plants release linalool, carveol, and nonane (2,2,4,4,6,8,8‐heptamethyl) under aphid stress.^[^ ^445^ ^]^ The cumulative levels of terpenoid volatiles increase with temperature (up to 30 °C).^[^ ^446^ ^]^ Additionally, increased release of methyl salicylate is observed in tomatoes infected with *Botrytis cinerea*.^[^ ^447^ ^]^ Therefore, monitoring plant health can be achieved by analyzing VOC emissions. Implementing reliable VOC monitoring can provide timely insights into plant growth status and enable responses to specific stresses, thereby mitigating potential economic losses.

Currently, many studies have utilized MEMS or optical sensors to monitor the release of VOCs from plants. For instance, Li et al. proposed a VSA based on piezoelectric cantilevers to monitor the release of (E)‐2‐hexenal, considered one of the biomarkers of plant late blight.^[^ ^367^ ^]^ They employed VSA to monitor (E)‐2‐hexenal in gas released from tomato leaves and potato tubers, and used ANN for data identification. The results showed that the VSA achieved an 89% accuracy in identifying plants affected by late blight. Sennik and colleagues developed an electronic nose system using CMUT arrays to selectively detect VOCs released by plants under different stress conditions (such as 1‐octanol, linalool, p‐xylene, styrene, and (*z*)‐3‐hexenol) (Figure 20c).^[^ ^108^ ^]^ To enhance selectivity, various materials including polymers, phthalocyanines, and metals were coated on the CMUT surface using inkjet printing and drop coating methods. The study found that the CMUT element coated with silver ink achieved a resolution of 3 ppb when exposed to 1‐octanol. The detection data were visualized using the K‐nearest neighbor (KNN) algorithm, achieving accurate gas classification with an accuracy exceeding 97%. In the realm of optical sensing, Shang et al. developed an LSPR sensor coated with AuNP‐containing molecularly imprinted sol–gel (MISG) for plant VOCs detection (Figure 20d).^[^ ^448^ ^]^ Under optimal conditions, the sensitivity of the AuNPs@MISG‐coated sensor was 12.33 times higher than that of a similar sensor without AuNPs. Subsequently, a multichannel optical sensing platform was constructed using AuNPs@MISG coated with different template molecules to detect plant VOCs in single and binary mixtures. Linear discriminant analysis, KNN, and naive Bayes classifier methods were employed to establish plant VOC recognition models. The results indicated that the KNN model had excellent potential for rapid and effective identification of plant VOCs (96.03%).

### 6.3 Applications of VOC Sensors in the Internet of Things

The IoT refers to a network composed of physical devices, vehicles, appliances, and other tangible objects embedded with sensors, software, and network connectivity, enabling them to collect and share data. Currently, physical sensors still dominate the applications of IoT. However, with technological advancements, we anticipate that VOC sensors will gradually increase their participation in IoT and become integral components of IoT sensor networks. VOC sensors are expected to contribute to various fields such as smart buildings, and food safety, environmental monitoring.

In smart buildings, VOC sensors combined with IoT technology provide important support for improving the health and safety of the built environment.^[^ ^449^, ^450^, ^451^ ^]^ A key application of VOC sensors in smart buildings is indoor air quality monitoring. Many building materials and furnishings release harmful gases such as formaldehyde and benzene, which can have serious effects on human health. By installing VOC sensors, smart building systems can monitor the concentration of VOCs in indoor air in real‐time. When high concentrations of harmful gases are detected, the system can automatically activate ventilation devices or air purifiers to promptly improve air quality. Yang et al. combined microfluidic chips with SERS spectra to develop an intelligent chip for detecting indoor pollutants (Figure 20e).^[^ ^452^ ^]^ The detection results demonstrate that this intelligent chip can accurately identify each component of VOC mixtures (formaldehyde, benzene/benzaldehyde, and cyclohexanone). This work contributes to advancing indoor air purification efforts.

The application of VOC sensors in food safety and quality control is increasingly widespread.^[^ ^453^, ^454^, ^455^ ^]^ By monitoring the release of gases during food production, storage, and transportation processes, they help ensure the freshness and safety of food. Researchers have successfully used SAW gas sensors to monitor different stages of ripeness in fruits, to assess the flavor quality of fruits.^[^ ^456^ ^]^ Artificial noses also play a significant role in food quality control. For instance, Leong et al. designed a machine learning‐driven “SERS taster” for flavor analysis of wines (Figure 20f).^[^ ^457^ ^]^ Four receptors were functionalized on the SERS substrate surface to introduce a wide range of receptor–flavor chemical interactions. Five representative wine flavors were tested, including higher alcohols (menthol), terpenes (linalool, limonene), and sulfur compounds (3‐mercaptohexyl acetate and 3‐mercapto‐1‐hexanol). The interactions between each receptor–flavor pair generated “SERS superprofile.” Subsequently, PCA and SVM were utilized to achieve clear flavor identification and quantification from these SERS superprofile. This work provides a potential paradigm shift for related applications such as food quality control and flavor analysis.

In addition to smart buildings and food safety, VOC sensors also demonstrate tremendous potential applications in multiple fields, such as environmental monitoring and exhaust emission detection.^[^ ^458^, ^459^, ^460^ ^]^ VOC sensors play a crucial role in environmental monitoring, particularly in the real‐time assessment of pollutants emitted from factories. Traditional environmental monitoring methods often require complex instruments and lengthy analyses, whereas artificial noses can rapidly and continuously detect VOCs and other pollutants in the air. Through the integration of IoT technology, these devices can be deployed at multiple monitoring points around factories, transmitting data in real‐time to help environmental protection agencies promptly understand and control pollution sources. The application of VOC sensors technology in exhaust emission detection provides new tools for environmental regulation. By monitoring vehicle exhaust emissions, environmental agencies can more effectively enforce emission standards and reduce urban air pollution. With continuous technological advancements, VOC sensors will play even greater roles in various fields, bringing more convenience and safety to society **Table** **2**.

**Table 2:** Comparison of performance and application areas of different sensors.

References	Device type	Working frequency	VOC receptors	VOCs type	Sensitivity	LOD	Response/recovery times	Detection range	Machine learning	Application
Ref. [374]	QCM array	21 MHz	PAN, PVAc, PVDF, PVP	Methanol, ethanol, propanol, butanol, benzene, toluene, and xylene	4.1 Hz mg L^−1^ (butanol); 0.6 Hz mg L^−1^ (methanol); 0.4 Hz mg L^−1^ (benzene)	–	6.3 s/9.3 s	1–40 mg L^−1^	LDA and SVM	–
Ref. [385]	FBAR array	2.45 GHz	PDMS@HKUST‐1	Water, methanol, ethanol, n‐propanol, n‐hexane, c‐hexane, and n‐heptane	0.82 × 10^3^ Hz ppm^−1^ (water), 1.27 × 10^3^ Hz ppm^−1^ (methanol), 0.83 × 10^3^ Hz ppm^−1^ (ethanol), 0.83 × 10^3^ Hz ppm^−1^ (n‐propanol), 0.28 × 10^3^ Hz ppm^−1^ (n‐hexane), 0.79 × 10^3^ Hz ppm^−1^ (c‐hexane), 0.53 × 10^3 ^Hz ppm^−1^ (n‐heptane)	2.34 ppm (water), 1.51 ppm (methanol), 2.31 ppm (ethanol), 2.31 ppm (n‐propanol), 6.85 ppm (n‐hexane), 2.43 ppm (c‐hexane), 3.62 ppm (n‐heptane)	–	20–100 ppm	–	–
Ref. [378]	8 cantilevers	20–150 kHz	Polyepichlorohydrin	Toluene, styrene, pentanal, octanal, hexanal, ethanol, 2‐methyl‐1‐propanol, butanol, benzadehyde, acetone, 6‐methyl‐5‐hepten‐2‐one, phenyl acetate, isopropanol	1.62 Hz ppm^−1^ (phenyl acetate)	7.5 ppm (phenyl acetate)	–	–	PCA	–
Ref. [379]	5 SAWs	–	Three types of odorant‐binding proteins	Octenol, carvone	20.8 Hz ppm^−1^ (octenol), 13.5 Hz ppm^−1^ (carvone)	0.48 ppm (octenol), and 0.74 ppm (carvone)	–	–	PCA	Food industry
Ref. [231]	CMUT	18.2 MHz	PIB	DMMP	37 ppb Hz^−1^	56 ppb	–	–	–	–
Ref. [136]	PMUT	2.58 MHz	GO	Water vapor	719 Hz per % RH	–	–	–	–	–
Ref. [362]	SEIRA	6–9 μm	–	Methanol, ethanol, and isopropanol	–	–	–	–	PCA, SVM	–
Ref. [395]	Waveguide	3.65–3.8 μm	–	Isopropyl alcohol (IPA) and acetone	7.069 × 10^−3^/‰ (IPA), 6.195 × 10^−3^/‰ (acetone)	191 ppm (IPA), 196 ppm (acetone)	–	1.5–18.4‰ (IPA), 2.1–23.75‰ (acetone)	CNN	IoT
Ref. [397]	SERS	–	SAMs	p‐Phenylenediamine (p‐PDA), 4‐aminophenylacetic acid (4‐APA), Rhodamine 6G (R6G) and folic acid (FA)	–	–	–	–	PCA	–
Ref. [432]	SERS	–	MOF	Benzaldehyde	–	0.19 ppb	–	–	PCA	Health monitoring
Ref. [289]	SERS	–	4‐mercaptobenzoate (MBA), 4‐mercaptopyridine (MPY), and 4‐aminothiophenol (ATP)	Methanol, ethanal, heptanal, octanal, and acetone	–	–	–	–	Partial least‐squares discriminant analysis (PLSDA) models	Health monitoring
Ref. [108]	CMUT array	4.7–4.9 MHz	Polymers, phthalocyanines, and metals	1‐Octanol, linalool, p‐xylene, styrene, and (*Z*)‐3‐hexenol	337 Hz ppm^−1^ (1‐Octanol)	≈ppm	–	–	KNN	Plant volatiles detection
Ref. [448]	LSPR	–	MISG	cis‐jasmone (CJ), α‐pinene, limonene, and γ‐terpinene	–	–	–	–	LDA, KNN	Plant volatiles detection
Ref. [452]	SERS	–	MXene, Ag–S	Aromatic compounds, aldehydes, ketones, or sulfides	–	≈ppb	–	–	PCA	Health monitoring, IoT

## 7 Conclusion

This review provides a comprehensive overview of the field of VOC sensing related to MEMS and optical technologies. Specifically, it describes the technical classification of VOC sensors, integrated sensing systems, and emerging applications. Advances in AI and machine learning have significantly promoted the development of VOC sensors, including electronic noses and photonic noses. These intelligent methods can classify and analyze complex data generated by sensor arrays. Despite the excellent performance of mass‐loading‐based electronic nose technology in certain applications, it still suffers from poor selectivity. To address this issue, researchers have proposed the concept of functionalized VOC receptors to enhance the selectivity of electronic noses. Additionally, by using cross‐reactive sensing techniques and machine learning algorithms, it is possible to quantify and identify multiple VOCs, thereby improving detection accuracy and reliability. In contrast, photonic nose technology achieves higher selectivity by utilizing spectral information related to absorption and scattering. However, the development of photonic noses faces unavoidable challenges, such as the complexity of optical systems and high equipment costs. These issues limit their wide application and popularization. Recently, the introduction of metasurfaces and tunable low‐dimensional materials brings new hope for the miniaturization, integration, and practicality of photonic noses, potentially driving broader applications in the future. Currently, VOC sensors are widely used in medical diagnostics, agricultural detection, food safety, and the IoT. Particularly in the medical diagnostics field, artificial olfactory sensor technology has garnered increasing attention. Developing efficient and reliable real‐time inspection techniques through breath analysis has become an essential tool for early noninvasive diagnostics, demonstrating significant potential.

Looking ahead, as technology advances and issues of reliability, standardization and miniaturization are resolved, it is anticipated that more VOC sensors will become part of our daily lives. Some foreseeable emerging applications include the IoT, smart home gas detection systems, wearable olfactory devices, and olfactory systems for robots. These applications require VOC sensors to become more integrated, intelligent, flexible, and adaptable. Furthermore, as the number of VOC sensor nodes increases, the exchange of large amounts of redundant data between sensing terminals and computing units will become a challenge. Therefore, developing in‐sensor computing technology will be a key direction for future development to improve data processing efficiency and system performance. In conclusion, although current technology shows broad application prospects, extensive research and technological innovation are still needed to bring these emerging VOC sensing applications to market and achieve wider application.

## Conflict of Interest

The authors declare no conflict of interest.

## Author Contributions

**Dongxiao Li**: Conceptualization (lead); Investigation (lead); Visualization (lead); Writing—original draft (lead). **Hong Zhou**: Conceptualization (lead); Investigation (lead); Visualization (lead); Writing—original draft (lead). **Zhihao Ren**: Conceptualization (equal); Investigation (equal); Visualization (supporting); Writing—original draft (supporting). **Chengkuo Lee**: Conceptualization (lead); Funding acquisition (lead); Supervision (lead); Writing—review & editing (lead). **Dongxiao Li** and **Hong Zhou** contributed equally to this work.
